# The Use of Central Nervous System Active Drugs During Pregnancy

**DOI:** 10.3390/ph6101221

**Published:** 2013-10-10

**Authors:** Bengt Källén, Natalia Borg, Margareta Reis

**Affiliations:** 1 Tornblad Institute, Lund University, Biskopsgatan 7, Lund SE-223 62, Sweden; 2 Department of Statistics, Monitoring and Analyses, National Board of Health and Welfare, Stockholm SE-106 30, Sweden; E-Mail: Natalia.Borg@socialstyrelsen.se; 3 Department of Medical and Health Sciences, Clinical Pharmacology, Linköping University, Linköping SE-581 85, Sweden; E-Mail: Margareta.Reis@liu.se.

**Keywords:** CNS active drugs, opioids, sedatives, hypnotics, antidepressants, psychostimulants, congenital malformations, neonatal morbidity

## Abstract

CNS-active drugs are used relatively often during pregnancy. Use during early pregnancy may increase the risk of a congenital malformation; use during the later part of pregnancy may be associated with preterm birth, intrauterine growth disturbances and neonatal morbidity. There is also a possibility that drug exposure can affect brain development with long-term neuropsychological harm as a result. This paper summarizes the literature on such drugs used during pregnancy: opioids, anticonvulsants, drugs used for Parkinson’s disease, neuroleptics, sedatives and hypnotics, antidepressants, psychostimulants, and some other CNS-active drugs. In addition to an overview of the literature, data from the Swedish Medical Birth Register (1996–2011) are presented. The exposure data are either based on midwife interviews towards the end of the first trimester or on linkage with a prescribed drug register. An association between malformations and maternal use of anticonvulsants and notably valproic acid is well known from the literature and also demonstrated in the present study. Some other associations between drug exposure and outcome were found.

## 1. Introduction

When the strong teratogenic effect of thalidomide was observed in 1961 [1,2] the interest was directed towards the possibility that other drugs could also harm the human embryo and a special interest was shown drugs which like thalidomide affected the central nervous system (CNS). One reason was that as these drugs could pass the blood-brain barrier they would be apt to pass the placental barrier and reach the embryo. Examples of early studies are the McBride study on imipramine and limb defects [3] and the Safra and Oakley study [4] on an association between the use of diazepam and cleft lip/palate. During the following decades the research methodology developed and large studies were made which did not rely on retrospective information on drug use. Much interest has been paid to anticonvulsants which were identified quite early as definite human teratogens [5] and more recently to antidepressant drugs, notably selective serotonin reuptake inhibitors (SSRI).

The following drug categories will be dealt with in this paper: opioids, anticonvulsants, drugs used for Parkinson’s disease, neuroleptics, sedatives/hypnotics, antidepressants, psychostimulants, and a group of other CNS active drugs. Among neuroleptics two drugs, dixyrazine and prochlorperazine, were treated separately from the other neuroleptics due to their specific use at nausea and vomiting of pregnancy (NVP). CNS-active drugs are sometimes used in combination, and some data on outcome after combined use of such drugs will be given.

## 2. Material and Methods with some General Information

The literature reviews presented try to bring up the most important literature within each field.

### 2.1. Use of CNS-Active Drugs during Early Pregnancy and Malformations in the Offspring

The data presented on outcome after maternal use of drugs are from the Swedish Medical Birth Register and cover the period 1996–2011. This register contains information on nearly all births in Sweden and is based on standardized medical records, used in the whole country [6]. These records contain four different components: one contains information from the first antenatal visit (usually during weeks 10–12), one contains information from the further antenatal care, one from the delivery, and one from the paediatric examination of the newborn—all newborns are examined by a qualified paediatrician. From this register, information was obtained on year of birth, maternal age at delivery, parity, smoking habits in early pregnancy, and body mass index (BMI).

At the midwife interview at the first antenatal care visit, the woman was asked if she had used any drugs since she became pregnant. The drug name was written down in clear text and was later centrally translated into Anatomical, Therapeutic, Chemical (ATC) codes. Information on dosage and pregnancy week when the drug was used was sometimes given, but this information is too imprecise to be useful.

Information on congenital malformations was obtained from the Medical Birth Register (MBR) but was supplemented with data from the Register of Birth Defects (RCM, previously Register of Congenital Malformations) and from a Hospital Discharge Register (HDR), containing diagnoses after inpatient treatments [7]. Linkage between the registers was made with the personal identification number which is unique for each Swedish resident. Data were given on any malformations and “relatively severe malformations” where a number of common and clinically little important conditions were excluded. These were: preauricular appendices, tongue tie, patent ductus at preterm birth, single umbilical artery, undescended testicle, unstable hip or hip (sub)luxation, and nevus. Also more specific malformation groups were analyzed when numbers were large enough, for instance, cardiovascular defects or hypospadias. A more extensive discussion of the health registers and the analytical tools can be found in [8].

During these years, 1,552,382 women gave birth—42,881 of them reported the use of at least one of the above-mentioned CNS active drugs in early pregnancy. Among all 1,575,847 infants born, 70,339 had any type of congenital malformation; 49,499 of them were classified as “relatively severe”, 16,145 had any cardiovascular defect, 11,157 of them had a ventricular septum defect (VSD) or an atrial septum defect (ASD), and 4,552 had hypospadias. The odds ratio (OR) with its 95% confidence interval (95% CI) for a specific malformation after maternal use of a specific drug (group) was estimated with Mantel-Haenszel methodology and the approximate confidence interval with Miettinen’s technique. When the expected number of exposed outcome was less than 10, a relative risk (RR) was calculated instead as the observed over expected number with 95% CI from exact Poisson distributions. In both situations, adjustment was made for year of birth, maternal age (5-year class), parity (1–4+), smoking in early pregnancy (unknown, none, <10 cigarettes/day, ≥10 cigarettes per day), and BMI (unknown, <18.5, 18.5–24.9, 25–29, 9. 30–34.9, ≥35).

### 2.2. General Information on Use of CNS-Active Drugs in Early Pregnancy

Table 1 gives an overview of the number of infants exposed in early pregnancy to drugs in each category. Three groups contain less than 1,000 individuals: drugs for Parkinsonism, psychostimulants, and “other CNS active drugs”.

**Table 1:** Overview of groups of CNS active drugs used in early pregnancy. Number of exposed infants.

Drug group	Number of infants	Per cent of CNS-active drugs	Pro mille of all infants
Opioids	7,780	18.1	4.9
Anticonvulsants	4,437	10.3	2.8
Drugs for Parkinson’s disease	167	0.4	0.1
Neuroleptics	4,113	9.6	2.6
Sedatives/hypnotics	7,222	16.8	4.6
Antidepressants	23,658	55.2	15.0
Psychostimulants	450	1.0	0.3
Other CNS active drugs	676	1.6	0.4

The sum of the groups exceeds the total number of exposed infants due to simultaneous use of drugs from different categories.

#### 2.2.1. Characteristics of Women Using CNS Active Drugs in Early Pregnancy

Table 2 compares some characteristics of women using CNS active drugs with those of other women. There is a clear-cut age dependency with increasing use at higher age, a lower use especially at parity 2, a strong association with smoking and BMI, with previous miscarriages, and with non-cohabitation. Women being born outside Sweden reported the use of these drugs more seldom than Swedish-born women did.

**Table 2:** Characteristics of women using CNS active drugs in early pregnancy. Each variable is adjusted for the other variables. Bold text marks statistical significance.

Variable	No with CNS drugs	Total number	OR	95% CI
*Maternal age*				
<20	664	27,828	**0.74**	**0.68–0.81**
20–24	5,289	210,254	**0.92**	**0.89–0.94**
25–29	11,911	485,407	1.00	Reference
30–34	14,431	531,265	**1.12**	**1.10–1.15**
35–39	8,608	248,516	**1.38**	**1.33–1.41**
40–44	1,919	47,018	**1.51**	**1.43–1.59**
≥45	85	2,054	**1.53**	**1.22–1.91**
*Parity*				
1	19,255	687,283	1.00	Reference
2	13,245	562,633	**0.78**	**0.76–0.80**
3	6,711	210,094	**0.95**	**0.92–0.98**
≥4	3,696	92,332	0.98	0.94–1.03
*Smoking*				
Unknown	782	95,377	-	-
None	33,748	1,316,102	1.00	Reference
<10 cigs/day	5,211	100,330	**2.30**	**2.17–2.30**
≥10 cigs/day	3,166	40,533	**3.50**	**3.37–3.63**
*BMI*				
Unknown	3,651	192,912	–	-
<18.5	980	33,624	**1.15**	**1.08–1.23**
18.5–24.9	21,306	841,302	1.00	Reference
25–29.9	10,618	336,114	**1.18**	**1.15–1.21**
30–34.9	4,185	106,013	**1.40**	**1.35–1.45**
≥35	2,159	42,377	**1.72**	**1.64–1.80**
Previous miscarriage				
Any	10,184	309,943	**1.08**	**1.05–1.10**
Two or more	2,723	74,240	**1.12**	**1.08–1.17**
Unwanted childlessness				
One year or more	3,586	114,889	0.98	0.95–1.02
Three years or more	1,473	45,480	0.98	0.93–1.04
Pre-existing diabetes	258	7455	1.07	0.94–1.22
Co-habitation				
Unknown	602	88,690	-	-
Co-habiting	37,263	138,323	1.00	Reference
Not co-habiting	5011	82,419	**1.91**	**1.05–1.93**
Mother’s country of birth				
Unknown	309	16,244	-	-
Sweden	36,810	1,235,542	1.00	Reference
Outside Sweden	5767	500,646	**0.60**	**0.58–0.62**

These effects varied somewhat between the different categories of drugs. The age dependency existed—more or less pronounced—for all categories except psychostimulants which showed an opposite association with a high use at young maternal age. Use of the two neuroleptics dixyrazine and prochlorperazine also deviated with a decreasing use after 30 years of age.

The use of opioids increased drastically with high parity and this was seen also for dixyrazine and prochlorperazine while other neuroleptics showed a low use at parity 3–4+.

The effect of smoking was seen in all groups, but was most pronounced for psychostimulants.

The association with high BMI was seen in all groups but neuroleptics (excluding dixyrazine and prochlorperazine).

The strength of the association with previous miscarriage varied – the only group for which this was not seen was dixyrazine and prochlorperazine.

For the total material, no association with unwanted childlessness was seen. For opioids, anticonvulsants and dixyrazine and prochlorperazine, a positive association was seen. For neuroleptics (excluding dixyrazine and prochlorperazine), sedatives/hypnotics and antidepressants a lower rate than expected of unwanted childlessness was seen.

A significant increase in the presence of pre-existing diabetes was only observed for anticonvulsants.

Non-cohabitation was increased in all drug groups—the highest rate was seen for psychostimulants. Only in the two neuroleptic groups, women born outside Sweden showed a higher drug use than Swedish-born women, for all other groups a lower use.

Table 3 shows the concomitant use of other drugs for all women who used CNS-active drugs. Practically all drug groups are used more often than among women not using CNS-active drugs; the only exceptions are gestagens and ovarian stimulation. The significance of this is of relevance foremost for drug groups which by themselves are associated with a known teratogenic activity. This is the case with antihypertensive drugs (notably for cardiovascular defects), possibly systemic corticosteroids (notably for cleft lip/palate), thyroid drugs, cytostatics and immunosuppressants. Antihypertensive drug use was associated with all CNS active drugs except for psychostimulants and “other” drugs. Use of systemic corticosteroids was notably associated with opioids, sedatives/hypnotics, antidepressants and “other drugs”. The use of thyroid drugs was not associated with opiates, dixyrazine and prochlorperazine, or “other drugs”. The few exposures for cytostatic drugs were mainly associated with opioids. Finally, immunosuppressant drugs were associated with opioids, anticonvulsants and antidepressants. In some analyses of congenital malformation risk infants exposed to concomitantly used drugs with teratogenic properties were excluded.

### 2.3. Use of CNS-Active Drugs during the 2nd or 3rd Trimester, Late Exposures

Drug exposure was determined by the use of the Swedish Register of Prescribed Drugs [9]. Since July 1, 2005, all prescriptions given in outpatient care are registered with the patient’s personal identification number (Id. number), date of buying the drug, ATC code and information on strength, dosage, and instructions for use. All prescriptions which referred to the CNS active drugs under study were selected. Using the Id. number, these records were linked to records of women who had given birth during 2006–2011 according to the Medical Birth Register. For each record, information was added on the ATC code and the number of days after the last menstrual period (LMP) when the drug was bought. This was calculated from the date of buying the drug, the date of delivery, and pregnancy duration in days, in most cases estimated by second trimester sonography.

**Table 3:** Concomitant use of other drugs by women using CNS active drugs. ORs adjusted for year of birth, maternal age, parity, smoking, and BMI. Bold text marks statistical significance

Drug group	With CNS-drugs	Total	OR	95% CI
Drugs for GERD	1,273	13,219	**3.23**	**3.05–4.42**
Aminosalicylic acid drugs	160	3,651	**1.55**	**1.32–1.82**
Folic acid	4,115	87,630	**1.53**	**1.40–1.58**
Antihypertensive drugs	564	5,510	**3.16**	**2.90–3.34**
Oral contraceptives	232	4,275	**1.87**	**1.64–2.14**
Gestagens	145	7,089	0.65	0.55–0.72
Ovarian stimulation	59	2,852	0.76	0.59–0.99
Systemic corticosteroids	310	5,419	**2.01**	**1.79–2.25**
Thyroid hormones	1,243	24,154	**1.63**	**1.54–1.93**
Antibiotics	1,366	38,778	**1.63**	**1.54–1.93**
Antimycotics	39	882	**1.65**	**1.20–2.28**
Cytostatics	16	143	**3.29**	**1.88–5.03**
Immunosuppressants	86	1,169	**2.41**	**1.94–3.00**
NSAID	1,719	23,226	**2.37**	**2.25–2.48**
Minor analgesics	5,098	105,391	**1.72**	**1.67–1.79**
Drugs for migraine	469	3,914	**4.56**	**4.16–5.00**
Drugs for rhinitis	1,719	18,155	**2.37**	**2.25–2.48**
Antiasthmatics	2,168	44,974	**1.62**	**1.55–1.70**
Antihistamines for allergy	1,064	21,989	**1.70**	**1.60–1.81**
Antihistamines for NVP	3,864	63,702	**2.43**	**2.34–2.51**

NSAID = non-steroid antiinflammatory drugs; NVP = nausea and vomiting in pregnancy.

Some maternal diagnoses in the Medical Birth Register were analyzed: preeclampsia/eclampsia (ICD-10 diagnoses O13-O15), gestational diabetes (O24.4), placenta abruption (O45), and haemorrhage around delivery (O46, O67 or O72).

From the same source, information on induction of delivery, gestation duration (in weeks), birth weight (in gram), infant sex, 7 min Apgar score, and neonatal diagnoses were obtained. The Apgar score and the neonatal diagnoses were used to identify a group of live born infants with neonatal morbidity as is shown in Table 4. Most analyses were made dichotomizing the data into with and without such neonatal morbidity but in some instances the various groups of diagnoses were analyzed separately. Such groups are shown in Table 5 with a separate listing for term infants. The marked excess in preterm infants is evident.

**Table 4:** Overview of presence of neonatal diagnoses among live born infants, 2006–2011, according to the Medical Birth Register (*n* = 650,009).

Diagnosis	ICD-10	Number	Per cent
Intrauterine asphyxia	P20	466	0.07
Birth asphyxia	P21	6,324	0.97
Respiratory distress of newborn	P22	20,839	3.21
Congenital pneumonia	P23	1,235	0.20
Neonatal aspiration syndrome	P24	894	0.14
Chronic respiratory disease originating perinatally	P27	751	0.12
Other respiratory conditions originating perinatally	P28	1,749	0.27
Intraventricular non-traumatic bleeding	P52	1,035	0.15
Jaundice	P58–P59	27,062	4.16
Hypoglycaemia	P70.4	14,538	2.24
Necrotic enterocolitis	P77	293	0.85
Neonatal convulsions	P90	1,081	0.17
Other CNS complications	P91	1,036	0.16
Feeding problems	P92	5,325	0.82
Apgar score at 5 min < 7	-	10,799	1.66
Any of the conditions listed above	-	64,971	10.00

**Table 5:** Comparison of some major groups of neonatal morbidity in live born infants and in the subgroup of term infants (*n* = 611,232, 94% of all).

Diagnosis		All infants		Term infants		
	ICD-10	Number	Per cent	Number	Per cent	Per cent preterm
Respiratory diagnoses	P21–P28	27,708	4.28	16,604	2.73	58.3
Apgar score at 5 min < 7	-	13,211	1.66	7,701	1.26	28.7
Jaundice	P58–P59	26,928	4.16	13,167	2.15	42.9
Hypoglycaemia	P70.4	14,455	2.24	9,961	0.23	19.5
CNS complications	P90–P91	1,776	0.27	1,429	0.23	19.5
Feeding problems	P92	5,325	0.82	3,467	0.57	28.7
Any of these conditions	-	64,971	10.0	43,122	7.06	33.6

Analysis was made using Mantel-Haenszel method to give odds ratios and approximate 95% confidence intervals were estimated using Miettinen’s method. Adjustment was made for year of birth, maternal age, parity, smoking status in early pregnancy and pre-pregnancy BMI (see above). Small for gestational age (SGA, <−2 SD) and large for gestational age (LGA, >2 SD) was determined from sex and parity specific growth curves [10].

### 2.4. General Information on the Use of CNS-Active Drugs after the First Trimester

Table 6 lists the number of women who filled prescriptions for the different types of CNS active drugs during the second or third trimesters. It should be noted that use during the 3rd trimester may well have occurred when the prescription was filled during the 2nd trimester so this dating does not necessarily mirrors the trimester of use. A total of 24,188 women with 24,631 infants were included. The total number of deliveries was 640,473 with 649,676 infants.

**Table 6:** Number of women filling prescriptions for various CNS active drugs during the 2nd or 3rd trimester and their infants.

Drug group	Women, 2nd trimester	Women, 3rd trimester	Total number of women	Total number of infants
Opioids	7,142	6,490	13,525	13,805
Anticonvulsants	642	670	1,216	1,231
Drugs for Parkinson	35	36	68	72
Neuroleptics except for dixyrazine or prochlorperazine	327	286	584	592
Dixyrazine or prochlorperazine	107	21	126	128
Sedatives or hypnotics	2,809	2,742	5,545	5,665
Antidepressants	4,053	4,334	7,940	8,053
Psychostimulants	85	103	180	184
Other CNS-active drugs	102	136	221	228

## 3. Results

### 3.1. Opioids

#### 3.1.1. Literature Review

Most early studies were performed by retrospective interviews and often referred to the use of codeine [11,12,13] but also studies on propoxyphene were published [14]. In the large prospective study from the Collaborative Perinatal Project in 1959 to 1965, 686 women reported the use of propoxyphene in early pregnancy (months 1–4) and 563 reported the use of codeine—no increased risk for congenital malformations was seen [15]. The only formally significant effect was seen for codeine on respiratory malformations based on eight observed against three expected cases.

More recently, a prospective investigation based on the Swedish Medical Birth Register studied outcome after a total of 4725 women who reported the use of opioids in early pregnancy and 2578 who had got it prescribed later in pregnancy [8]. The majority had used dextropropoxyphene followed by codeine combined with paracetamol or acetylsalicylic acid (ASA). The risk for any relatively severe malformation was slightly increased but statistical significance was not reached (OR = 1.13, 95% CI 0.97–1.31). For individual drugs, only buprenorphine reached formal statistical significance (RR = 2.99, 95% CI = 1.10–6.50) but this was based on only six exposed infants with malformations among a total of 66 exposed. Codeine was also used as an antitussive but the number of exposures was low, only 34 during the first trimester and 22 during later pregnancy.

The question on the possible teratogenic effect of codeine was investigated in a study based on the Norwegian Mother and Child Cohort [16]. Ascertainment of exposure data was obtained by questionnaires during pregnancy, the first one given during weeks 17–18. The total response rate was only 43.5%. Data referred to 72,934 women—among them 1,693 reported the use of codeine during the first trimester and 2,666 during any part of the pregnancy. Forty infants exposed during the first trimester had a major congenital malformation (OR = 0.8, 95% CI 0.5–1.1). The study showed a high use of NSAIDs, antidepressants, anxiolytics, hypnotics and antipsychotics together with codeine.

In 2011, a paper was published from the US National Birth Defects Prevention Study describing a case-control study of maternal opioid analgesics and birth defects [17]. It found a statistically significant association between the reported use of opioids and some of the many congenital malformations studied: conoventricular septal defects, atrioventricular septal defects, hypoplastic left heart syndrome, spina bifida, and gastroschisis. Separate studies were made for codeine, hydrocodone, oxycodone, and meperidine but numbers of exposed cases were then small and possible differences between specific drugs could well be random. The results of the study are uncertain because of the retrospective method of drug use ascertainment and the high non-response rate (about 30%).

A review article on tramadol in pregnancy [18] stressed the lack of information on tramadol use in early pregnancy but quoted the Abstract of a prospective French TIS study [19] on 146 exposed pregnancies and 292 matched controls which did not demonstrate a difference in malformation rate, based on six cases in the exposed and 15 in the non-exposed group. Infants born of mothers who abuse opiates (e.g., heroin) during pregnancy show numerous neonatal deviations with a high rate of preterm births and neonatal death [20]. A high percentage of the infants show neonatal abstinence syndrome (NAS) which can be quantified by the Finnegan score [21]. NAS is caused by withdrawal of opiates in the newborn and is associated with dysfunction of the central and autonomic nervous system, the gastrointestinal tract and the respiratory system. Also after medical use of opioid analgesics, an increased risk for NAS has been described for tramadol [22], propoxyphene [23] and codeine [24]. An increased risk for preterm birth and neonatal complications was found after maternal use of opioids, less marked after dextropropoxyphene or codeine than after other opioids [25].

#### 3.1.2. Data from the Swedish Medical Register

**Figure 1:**
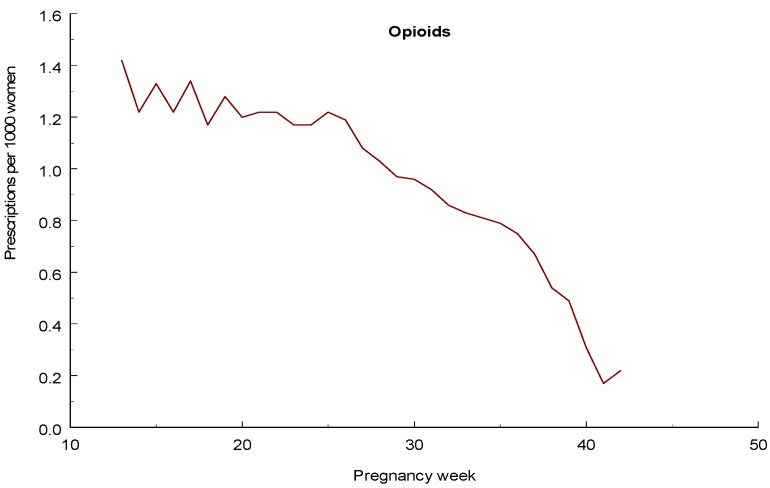
Number of women who had filled a prescription for an opioid for each gestational week.

During the years 1996–2011 a total of 7,654 women reported the use of an opioid in early pregnancy and they had 7,780 infants. The distribution of the different drugs is shown in Table 7. The largest number of exposures was seen for dextropropoxyphene, codeine, and tramadol. The Table also shows the specific opioids prescribed during the 2nd and 3rd trimesters. A total of 13,525 women with 13,805 infants had filled prescriptions on opioids during the 2nd or 3rd trimester. Figure 1 shows the weekly rates (per 1,000 women pregnant in that week) of any opioid prescription during these trimester. It can be seen that up to about week 25, only a moderate decline in prescription rate occurred but then the rate dropped markedly.

Table 8 summarizes the presence of malformations among infants whose mothers had used opioids in early pregnancy. An increased risk for any malformations or “relatively severe malformations” is seen but the only specific malformation which is increased is pes equinovarus.

**Table 7:** Number of children exposed in early pregnancy for opioids and number of children born to women who filled prescriptions during the 2nd or 3rd trimester (late exposures).

ATC	Drug name	Early exposures	Late exposures
N02AA01	morphine	253	55
N02AG01	morphine+spasmolytics	185	653
N02AA03	hydromorphone	3	3
N02AG04	hydromorphone+spasmaolytics	21	1
N02AA05	oxycodon	54	129
N02AA59	codeine+paracetamol	2,306	6,971
N02AB01	ketobemidone	69	86
N02AG02	ketobemidone+spasmolytica	192	33
N02AB02	pethidine	24	1
N02AB03	fentanyl	7	9
N02AC02	methadone	41	-
N02AC04	dextropropoxyphene	1,972	5,932
N02AC54	dextropropoxyphene+paracetamol/ASA	1,426	-
N02AD01	pentazocine	5	-
N02AE01	buprenorphine	86	-
N02AX02	tramadol	1,603	1,254
N02A	unspecified opioid	22	-

**Table 8:** Congenital malformations among infants exposed to opioids in early pregnancy. ORs calculated if three or more outcomes. Bold text marks statistical significance.

Malformation	Number With drug	Total number	OR/RR	95% CI
All	401	70,339	1.02	0.92–1.12
Relatively severe	282	48,499	1.03	0.91–1.15
Chromosome anomaly	15	2,933	0.83	0.50–1.37
Neural tube defects	5	734	1.12	0.36–2.60 #
Other CNS malformations	8	1,131	1.40	0.60–2.76 #
Severe eye malformations	1	579	-	-
Severe ear malformations	1	280	-	-
Orofacial clefts	8	2,756	0.49	0.25–0.96
Any cardiac defect	97	16,145	1.04	0.85–1.27
Septal cardiac defect	67	11,157	1.04	0.82–1.32
Oesophageal atresia	1	445	-	-
Small gut atresia	1	392	-	-
Anal atresia	2	590	-	-
Pyloric stenosis	6	1,101	0.92	0.34–2.01 #
Abdominal wall defect	3	413	1.44	0.30–4.19 #
Diaphragmatic hernia	3	368	1.36	0.28–3.99 #
Hypospadias	25	4,552	0.97	0.65–1.44
Major renal malformations	3	882	0.58	0.12–1.71 #
Pes equinovarus	22	2,127	**1.68**	**1.10–2.55**
Poly- or syndactyly	16	3,084	0.95	0.58–1.56
Limb reduction defects	8	830	1.73	0.75–3.41 #
Craniostenosis	3	862	0.60	0.12–1.76 #

# RR from observed/expected numbers with exact 95% CI based on Poisson distributions.

Table 9 specifies data for the most often used opioids. The only drug which reaches statistical significance is tramadol for pes equinovarus. The estimates for dextropropoxyphene and tramadol do not differ significantly, however. The OR for pes equinovarus after tramadol increases slightly when concomitantly used drugs with a possible teratogenic effect (notably anticonvulsants) were excluded (RR = 3.88, 95% CI 1.86–7.13) and even more if also women with previous miscarriages and/or born outside Sweden were removed (RR = 4.17, 95% CI 1.35–9.72) leaving only five infants with pes equinovarus.

**Table 9:** Congenital malformations among infants exposed in early pregnancy for three specific opioids. Codeine +paracetamol; dextropropoxyphene, possibly with paracetamol or ASA, and tramadol. Bold text marks statistical significance.

Malformation	Drug	Number with drug	OR/RR	95% CI
Relatively severe	codeine	81	1.10	0.88–1.37
	dextropropoxyphene	114	1.10	0.88–1.37
	tramadol	63	1.15	0.89–1.49
Any cardiac defect	codeine	28	1.08	0.74–1.56
	dextropropoxyphene	34	0.80	0.58–1.12
	tramadol	23	1.29	0.85–1.95
Hypospadias	codeine	10	1.34	0.72–2.50
	dextropropoxyphene	9	0.80	0.42–1.55
	tramadol	5	0.99	0.32–2.31 #
Pes equinovarus	codeine	4	1.08	0.29–2.75 #
	dextropropoxyphene	9	1.47	0.67–2.80 #
	tramadol	10	**3.60**	**1.72–6.62 #**

# RR from observed/expected numbers with exact 95% CI based on Poisson distributions.

Table 10 shows outcome after exposure to opioids during the 2nd or 3rd trimester. There is a significantly increased risk for preeclampsia, placenta abruption, and haemorrhage around delivery. For preeclampsia there was no major difference between the three large opioid groups: codeine, dextropropoxyphene, and tramadol. None of these drugs was associated with an increased risk of gestational diabetes. The association with placenta abruption was not seen for codeine (OR = 1.10, 95% CI 0.77–1.56) but for dextropropoxyphene (OR = 1.68, 95% CI 1.20–2.29) and for tramadol (OR = 1.82, 95% CI 1.62–3.23). A strong association with haemorrhage was seen for dextro-propoxyphene (OR = 1.93, 95% CI 1.79–2.08), a weaker for codeine (OR = 1.20, 95% CI 1.10–1.38) and none for tramadol (OR = 1.07, 95% CI 0.86–1.32).

**Table 10:** Outcomes after maternal use of opioids during the 2nd or 3rd trimester. Bold text shows statistical significance.

Outcome	With opioid	In population	OR	95% CI
Preeclampsia	743	25,688	**1.34**	**1.24–1.44**
Gestational diabetes	196	7,118	1.04	0.90–1.20
Abruption of placenta	70	2,379	**1.37**	**1.09–1.73**
Haemorrhage around delivery	1,408	46,981	**1.48**	**1.30–1.57**
Induction of delivery	2,659	81,793	**1.61**	**1.54–1.68**
Preterm birth < 32 weeks	86	31,394	0.74	0.60–0.92
Preterm birth < 37 weeks	764	20,712	**1.12**	**1.03–1.20**
Low birth weight < 2500 g	482	16,440	1.04	0.95–1.15
Small for gestational age (SGA)	326	19,745	0.93	0.82–1.02
Large for gestational age (LGA)	591	67,057	**1.30**	**1.19–1.42**
Neonatal diagnosis	1,630	27,708	**1.15**	**1.10–1.22**
Respiratory diagnosis	740	14,455	**1.29**	**1.20–1.39**
Hypoglycaemia	391	25,410	**1.14**	**1.03–1.26**
Jaundice	597	26,928	0.96	0.88–1.04
CNS diagnoses	170	6,940	**1.19**	**1.02–1.38**
Low 5 min Apgar score	314	13,211	**1.11**	**1.00–1.25**

There is a significantly increased risk for preterm birth (<37 weeks) but not for very preterm birth (<32 weeks). This effect is less pronounced for dextropropoxyphene (OR = 1.08, 95% CI 0.90–1.21), intermediate for codeine (OR = 1.17, 95% CI 1.00–1.30) and strongest for tramadol (OR = 1.30, 95% CI 1.04 − 1.62) but these three ORs may all be estimates from the common OR.

There is no effect on low birth weight or SGA but an increased rate of LGA. This was strongest for dextropropoxyphene (OR = 1.46, 95% CI 1.29–1.65) , intermediate for codeine (OR = 1.25, 95% CI 1.10–1.40), and absent for tramadol (OR = 1.05, 95% CI 0.78–1.41).

The risk for neonatal morbidity was increased, notably for respiratory diagnoses and CNS diagnoses. This risk was higher for tramadol (OR = 1.31, 95% CI 1.12–1.54) than for codeine (OR = 1.17, 95% CI 1.09–1.26) and for dextropropoxyphene (OR = 1.15, 95% CI 1.07–1.25). The risk estimates were high for morphine, oxycodone and ketobemidone but numbers were low and only for ketobemidone was formal statistical significance reached (OR = 2.30, 95% CI 1.49–3.55, based on 24 cases).

Use of opioids in early pregnancy seems not to be associated with any definite teratogenic effect. The only association seen is with pes equinovarus and this association might be stronger for tramadol than for other opioids but this finding needs verification.

Use of opioids in the late part of pregnancy is associated with a number of pregnancy and neonatal complications. There seems to be some variability in effects between different opioids which speaks for a drug effect. Another possible explanation is that these drugs could be prescribed because of complications during pregnancy which may lead to early induction of delivery and effects on the fetus. Most convincing are perhaps the increased risks of respiratory problems and of various CNS diagnoses.

### 3.2. Anticonvulsants

#### 3.2.1. Literature Review

The first study of anticonvulsant use during pregnancy in 1968 found no increased risk for congenital malformations—it was based on 262 exposed infants [26]. Meadow [5], however, found that mothers of infants with orofacial clefts had epilepsy in a higher rate than expected and suggested an association. Since then, numerous studies have verified the observation that women who use anticonvulsants have an increased risk to have a malformed infant and many reviews have been published on the subject (e.g., [27,28]). Among the first generation anticonvulsants (introduced in Europe before 1980), an increased risk for congenital malformations was seen for nearly all drugs but specifically for valproic acid, the use of which was linked with a markedly increased risk for spina bifida [29]. Valproic acid has later been shown to carry a higher teratogenic risk than other anticonvulsants. Another early lesson was that monotherapy carried less risk for teratogenesis than polytherapy [30,31].

Maternal use of anticonvulsive drugs also increases the risk for minor abnormalities like facial and digital abnormalities. This was first described by Béthenod and Frédérich [32] (mainly after phenobarbital) and by Hanson *et al*. [33] after hydantoid but has later been identified also after some other anticonvulsants.

A number of large registers are operating specifically on anticonvulsants during pregnancy: the UK and Ireland Epilepsy and Pregnancy Register (e.g., [34,35]), the North American AED Pregnancy Registry [36], the Australian Pregnancy Register (e.g., [37]), and the EURAP Epilepsy and Pregnancy Register, mainly from Europe [38]. Pregnancy registers for specific anticonvulsants have also existed, e.g., for gabapentine [39] and lamotrigine [40]. The Neurodevelopment Effects of Antepileptic Drugs (NEAD) study mainly concerns neurodevelopmental effects of anticonvulsants [41].

These registers in most cases have no control material but consist of the prospective collection of pregnancies exposed to anticonvulsants. Outcomes are often identified from questionnaires to reporting doctors or to patients. Their greatest value lies in comparisons between different anticonvulsant therapies.

Other studies have made use of national health registers. A Danish study was based on prescription data [42] and a Norwegian one used the Norwegian Medical Birth Registry [43]. Data from the Swedish Medical Birth Register have been published [8,44] and a Finnish study has also been published [45]. These studies permit comparisons with unexposed infants and are based on malformation ascertainment from various health registers.

The general effects of anticonvulsant exposure during early pregnancy are relatively well known. At present, studies mainly concentrate on the effects of the second-generation anticonvulsants, notably lamotrigine, topiramate, gabapentine, and levetiracetam.

*Lamotrigine* is the best studied one among the newer anticonvulsants but the results vary between different studies. Most have found no increased risk for major congenital malformations [8,36,40,42] while one study [38] found a dose-dependent increase of the malformation rate (2% at <300 mg/day and 4.5% at ≥300 mg/day) which agrees with other findings [34] where the OR at doses >200 mg/day was 5.4 (95% CI 3.3–8.7). In most studies, the number of exposed infants was relatively low which is also seen from the wide confidence intervals. A specific relationship between lamotrigine exposure and orofacial clefts in the infant has been suggested [46]. An analysis of the adverse event reporting system of FDA [47] also suggested an association between lamotrigine use and jaw and oral malformations. An analysis of data from the EUROCAT found no increased risk for orofacial clefts after lamotrigine exposure but the confidence interval was wide: OR = 0.67, 95% CI 0.10–2.34 [48].

*Topiramate* has also been the subject of a number of investigations. Two relatively small studies were published in 2008 [49,50]. In one of them [50], 9% of the 70 infants had a major congenital malformation and four of them were orofacial clefts. Among 108 infants exposed to topiramate, five were malformed (OR = 1.44, 95% CI 0.58–3.58) [41] and another study found 15 malformed infants among 359 exposed with an RR of 2.2 (1.2–4.0) and noticed an increased risk for cleft lip [35]. The total malformation rate after topiramate exposure in monotherapy was 4.33% against 3.77% in unexposed infants but no specific effect on orofacial clefts was seen [51]. In retrospectively collected data an OR of 5.4 (95% CI 1.5–20.1) for cleft lip/palate was found after topiramate exposure [52].

*Levetiracetam* has been studied only in small series of exposures. A review of the literature identified 147 exposed infants with 2% major and 4.8% minor malformations [53]. Among 58 exposed infants none had a malformation [42]. In a larger material of 450 exposed infants, 11 had malformations [36]. Among 22 pregnancies exposed for this drug, none of the infants was malformed [37]. In the most recent study on 304 monotherapy and 367 polytherapy exposures two and 19 malformed infants were found, respectively [54].

*Other second generation anticonvulsants*. Only very small studies exist on other second generation anticonvulsants. A report on gabapentine [39] from a Gabapentine Pregnancy Register stated that among 44 live births exposed to this drug (1/3 in monotherapy), two had major malformations.

Most data on the teratogenicity of anticonvulsants are based on cases with epilepsy. Recently these drugs have had a use in other types of patients, e.g., as mode stabilizers at bipolar disease. It is not certain that the drug have similar effects in such circumstances as often dosage is lower.

Already in the review by Bossi [55] in 1983, effects of maternal use of anticonvulsants on the neonate were quoted: an increased risk for intrauterine growth retardation and for decreased head circumference.

An increased risk of preterm birth, low birth weight and intrauterine growth retardation was found after maternal use of anticonvulsants and also of neonatal hypoglycaemia, respiratory complications, low Apgar score and CNS diagnoses [8]. Similar findings were reported from Norway [43]. An increased risk for preterm birth and for neonatal complications was also found [25]. In a study comparing the neonatal effects of different anticonvulsants in monotherapy, the strongest effect on intrauterine growth retardation was seen from valproic acid and carbamazepine with roughly normal values for lamotrigine and phenytoin [56]. Similar differences were seen for small head circumference which was the case also in the study by Almgren *et al*. [57].

#### 3.2.2. Data from the Swedish Medical Register

**Figure 2:**
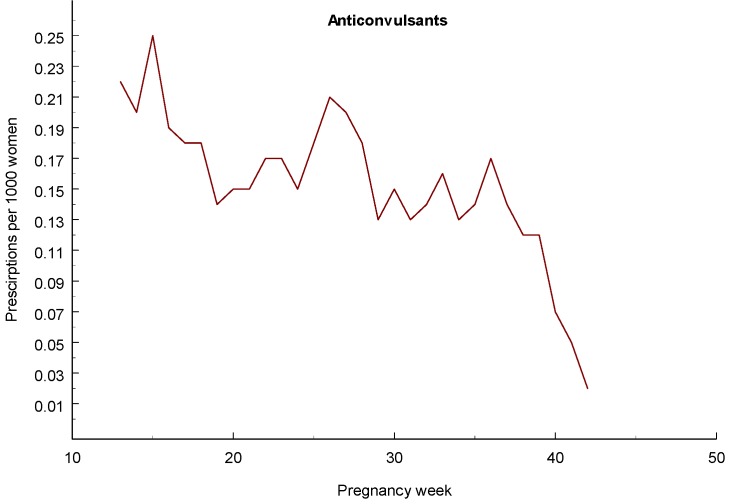
Diagram showing number of women per 1,000 who had filled a prescription for an anticonvulsant for each gestational week.

Table 11 summarizes the reported use of anticonvulsants in early pregnancy, a total of 4,290 women with 4,437 infants, and prescriptions of anticonvulsants during the 2nd or 3rd trimester, a total of 1 216 women with 1,231 infants. In the majority of cases (1,079), only one anticonvulsant was prescribed during this period and in 152 there was polytherapy (use of more than one antivonvulsant). The rate of prescriptions per pregnancy weak declines to about half (Figure 2).

**Table 11:** Number of children exposed to anticonvulsants in early pregnancy and children of women who filled prescriptions for anticonvulsants during the 2nd or 3rd trimester divided into specific drugs.

ATC code	Drug name	Early exposures	Late exposures
N03AA02	phenobarbital	28	3
N03AA03	primodone	12	3
N03AB02	phenytoin	173	11
N03AD01	ethosuximide	30	2
N03AE01	clonazepam	173	44
N03AF01	carbamazepine	1,706	285
N03AF02	oxcarbazepine	58	24
N03AG01	valproic acid	862	212
N03AG04	vigabatrin	29	1
N03AX09	lamotrigine	1,337	487
N03AX11	topiramate	102	51
N03AX12	gabapentin	143	64
N03AX14	levetiracetam	151	74
N03AX15	zonisamide	7	4
N03AX16	pregabalin	-	128

Table 12 summarizes the presence of congenital malformations in infants exposed to anticonvulsants in early pregnancy. There is a general and statistically significant risk increase for any malformation, for “relatively severe malformations”, other CNS defects, orofacial clefts, congenital heart defects, diaphragmatic hernia, hypospadias, and pes equinovarus, but risk increases were also seen for neural tube defects, alimentary tract atresia or stenosis, severe renal malformations and craniostenosis even though statistical significance was not reached. If all alimentary tract atresia/stenosis were analyzed together, use of any anticonvulsant was associated with RR = 2.05 (95% CI 1.17–3.32).

**Table 12:** Presence of congenital malformations in infants whose mothers used anticonvulsants in early pregnancy. Bold text marks statistical significance.

Malformation	Number with drug	Total number	OR/RR	95% CI
All	334	70,339	**1.49**	**1.34–1.66**
Relatively severe	239	48,499	**1.55**	**1.36–1.76**
Chromosome anomaly	7	2,933	0.73	0.29–1.50
Neural tube defects	4	734	1.59	0.43–4.08 #
Other CNS malformations	10	1,131	**3.02**	**1.51–5.27 #**
Severe eye malformations	1	579	-	-
Severe ear malformations	0	280	-	-
Orofacial clefts	20	2,756	**2.10**	**1.35–3.26**
Any cardiac defect	89	16,145	**1.71**	**1.39–2.10**
Septal cardiac defect	65	11,157	**1.65**	**1.31–2.10**
Oesophageal atresia	3	445	2.33	0.48–6.80 #
Small gut atresia	4	392	3.57	0.97–9.14 #
Anal atresia	5	590	2.94	0.96–6.86 #
Pyloric stenosis	7	1,101	1.79	0.72–3.71 #
Abdominal wall defect	2	413	-	-
Diaphragmatic hernia	5	368	**3.82**	**1.24–8.41 #**
Hypospadias	39	4,552	**2.65**	**1.95–3.59**
Major renal malformations	5	882	1.88	0.61–4.39 #
Pes equinovarus	17	2,127	**2.52**	**1.47–4.03 #**
Poly- or syndactyly	17	3,084	**1.75**	**1.09–2.81**
Limb reduction defects	3	830	1.02	0.21–2.95 #
Craniostenosis	5	862	1.84	0.60–4.29 #

# RR from observed/expected numbers with exact 95% CI based on Poisson distributions.

The risk for “relatively severe malformations” was significantly higher after polytherapy than after monotherapy: OR = 3.17 (95% CI 2.40–4.19) and OR = 1.37 (95% CI 1.19–1.58), respectively. This difference was seen both with and without valproic acid. Monotherapy without valproic acid showed a low OR, 1.13 (95%CI 0.94–1.35) while polytherapy without valproic acid showed a significantly increased OR, 2.10 (95% CI 1.46–3.02). The corresponding values for valproic acid was 2.30 (95% CI 1.82–2.91) for monotherapy and 4.98 (95% CI 3.40–7.30) for polytherapy. In both situations, polytherapy carried a significantly higher risk than monotherapy.

Table 13 shows the number of infants exposed to specific anticonvulsants in early pregnancy. Total numbers and numbers of infants exposed in monotherapy (no other anticonvulsant) are given. The Table also gives number of infants with “relatively severe malformations” after exposure to specific anticonvulsants in monotherapy. The lowest risk appeared to be for carbamazepine followed by lamotrigine but the upper confidence limits were 1.35 and 1.50, respectively. For many drugs the confidence intervals were large and did not exclude 1.0.

Topiramate in monotherapy resulted in a significantly increased “relatively severe malformation” risk based on six exposed cases. One of these had Down syndrome, two had polydactyly, one pyloric stenosis, one coarctation of aorta, and one was multimalformed with cleft lip/palate, transposition of the great vessels and hypospadias.

**Table 13:** Number of infants exposed in early pregnancy to various anticonvulsants, total and as monotherapy. Number with “relatively severe malformations” after monotherapy. Bold text marks statistical significance.

ATC	Drug name	Total number	Mono- therapy	Number malformed	OR/RR	95% CI
N03AA02	phenobarbital	28	17	2	-	-
N03AA03	primidone	12	9	0	-	-
N03AB02	phenytoin	173	140	12	1.84	0.95–3.21 #
N03AD01	ethosuximide	30	12	1	-	-
N03AE01	clonazepam	173	106	5	1.31	0.42–3.05 #
N03AF01	carbamazepine	1,706	1,511	58	1.04	0.80–1.35
N03AF02	cxcarbazepine	58	40	4	2.27	0.62–5.82 #
N03AG01	valproic acid	862	697	62	**2.30**	**1.82**–**2.91**
N03AG04	vigabatrin	29	8	0	-	-
N03AX09	lamotrigine	1,337	1,084	37	1.08	0.78–1.50
N03AX11	topiramate	102	49	6	**3.73**	**1.97**–**8.11 #**
N03AX12	gabapentine	143	119	2	-	-
N03AX14	levetriacetam	151	57	1	-	-
N03AX15	zonisamide	7	3	1	-	-
N03AX16	pregabalin	128	111	2	-	-

# RR from observed/expected numbers with exact 95% CI based on Poisson distributions.

In Table 14 the three most common anticonvulsants in monotherapy were studied for six specific malformation groups where numbers were enough to permit analysis. A significant risk increase was seen for all conditions except poly/syndactyly after exposure to valproic acid: the strongest effect was seen for hypospadias followed by pes equinovarus. Among the five cases of spina bifida, all were exposed to valproic acid, one of them also with carbamazepine.

**Table 14:** Effect of use of three specific anticonvulsants in monotherapy on the occurrence of six groups of malformations. Bold text marks statistical significance. N values mark number of infants in population with this type of malformation.

Malformation	Anticonvulsant	Number with Drug	OR/RR	95% CI
Orofacial cleft	carbamazepine	4	1.26	0.34–3.23 #
(*n* = 2756)	valproic acid	5	3.33	1.08–7.78 #
	lamotrigine	4	2.14	0.58–5.48 #
Any cardiac defect	carbamazepine	13	0.70	0.41–1.21
(*n* = 16,145)	valproic acid	23	2.67	1.68–4.04 #
	lamotrigine	10	0.81	0.44–1.50
Septum defect	carbamazepine	11	0.81	0.48–1.55
(*n* = 11,155)	valproic acid	17	2.66	1.55–4.27 #
	lamotrigine	14	1.52	0.83–2.55 #
Hypospadias	carbamazepine	5	0.96	0.31–2.24 #
(*n* = 4552)	valproic acid	20	7.33	4.48–11.3 #
	lamotrigine	3	0.87	0.18–2.04 #
Pes equinovarus	carbamazepine	5	2.23	0.72–5.21 #
(*n* = 2127)	valproic acid	6	6.12	2.25–13.3 #
	lamotrigine	1	-	-
Poly/syndactyly	carbamazepine	5	1.45	0.47–3.38 #
(*n* = 3084)	valproic acid	1	-	-
	lamotrigine	3	1.33	0.27–3.90

# RR from observed/expected numbers with exact 95% CI based on Poisson distributions.

Outcomes after maternal use of anticonvulsants during the 2nd or 3rd trimester are shown in Table 15. There was no significant effect on maternal diagnoses except for delivery inductions. In a larger material [8], a significantly increased risk for preeclampsia, placental abruption, and intrapartum haemorrhage was found.

**Table 15:** Outcomes after maternal use of anticonvulsants during the 2nd or 3rd trimester. Bold text marks statistical significance.

Outcome	With anti–convulsant	In population	OR	95% CI
Preeclampsia	63	25,688	1.18	0.91–1.53
Gestational diabetes	14	7,118	0.87	0.81–1.45
Abruption of placenta	5	2,379	1.07	0.35–2.50 #
Haemorrhage around delivery	106	46,981	1.19	0.97–1.45
Induction of delivery	240	81,793	**1.56**	**1.36–1.80**
Preterm birth < 37 weeks	88	31,394	1.34	0.95–1.89
Low birth weight < 2500 g	53	20,712	1.27	0.97–1.69
Small for gestational age	41	16,440	1.25	0.91–1.72
Large for gestational age	41	19,745	1.02	0.75–1.40
Neonatal diagnosis	157	67,057	**1.24**	**1.05–1.46**
Respiratory diagnosis	72	27,708	**1.39**	**1.10–1.76**
Hypoglycaemia	45	14,455	**1.51**	**1.12–2.02**
Jaundice	46	26,928	0.83	0.62–1.12
CNS diagnoses	31	6,940	**2.39**	**1.68–3.39**
Low 5 min Apgar score	34	13,211	1.34	0.95–1.09

# RR from observed/expected numbers with exact 95% CI based on Poisson distributions.

There was a significantly increased risk for preterm birth, slightly higher after polytherapy (OR = 1.80, 95% CI 1.03–3.13) than after monotherapy (OR = 1.39, 95% CI 1.09–1.71) but this difference may be random. A corresponding increase in low birth weight and SGA infants was not statistically significant but it was in a larger material [8]. The significantly increased risk for neonatal morbidity seemed to be due both to respiratory problems and CNS symptoms. It was only slightly higher after polytherapy (1.52) than after monotherapy (1.25) and this difference may be random.

When individual anticonvulsants were studied, valproic acid was associated with an increased risk for preterm birth (OR = 1.61, 95% CI 1.16–2.98) while the ORs for carbamazepine (1.22, 95% CI 0.95–2.00) and lamotrigine (OR 1.21, 95% CI 0.83–1.77) were lower and did not reach statistical significance. The same phenomenon was seen for neonatal morbidity. The risk after valproic acid (OR = 1.99, 95% CI 1.43–2.78) was higher than for carbamazepine (OR = 1.00, 95% CI 0.69–1.43) or lamotrigine (OR = 1.11, 95% CI 0.81–1.42) and this difference seemed not be random. There was no difference in effect of valproic acid in monotherapy and in polytherapy.

Maternal use of anticonvulsants is associated with an increased risk of congenital malformations in the offspring. This is specifically pronounced for valproic acid where a strong association exists with neural tube defects, orofacial clefts, cardiac defects, hypospadias and pes equinovarus. Anticonvulsants in monotherapy show a lower risk than such drugs in polytherapy, not statistically significant for carbamazepine or lamotrigine,. If possible, polytherapy should be avoided and valproic acid should be used only when no suitable alternative exists.

Use of anticonvulsants during the 2nd or 3rd trimester was associated with rather small pregnancy and neonatal effects. There were some weak evidence for a larger risk after polytherapy than after monotherapy but the differences were not large. Valproic acid had a markedly higher effect on preterm birth and on neonatal morbidity than carbamazepine and lamotrigine – these three drugs were the only ones with enough number of exposures to permit drug-specific analyses. In previous studies an effect on head circumference could be seen in the newborn for valproic acid and carbamazepine but not for lamotrigine [56,57]. The effects of anticonvulsant use in late pregnancy seems not to have any major influence on the perinatal outcome but if valproic acid can be avoided, that seems to be of some benefit.

### 3.3. Drugs Used for Parkinson’s Disease

#### 3.3.1. Literature Review

The drugs, classified as drugs used at Parkinsonism, are also used for other purposes, e.g., treatment of restless legs. Relatively little is known about their use during pregnancy. Amantadine has been used at Parkinson’s disease but also for prevention of influenza. A few case reports linked maternal use of this drug with severe congenital malformations in the offspring [58,59]. Among four women who had used amantadine for Parkinsonism, two gave birth and one of the infants had an inguinal hernia [60]. The teratogenic property of this drug is thus uncertain. For other drugs used at Parkinsonism, no certain signs of teratogenicity exist but this is mainly based on case reports. The only exception is bromocriptine which is also used to restore fertility in hyperprolactinaemic women. In this setting, a follow-up of 2,587 pregnancies was made without the identification of any ill effects on the offspring [61]. Cabergoline is used for Parkinsonism but also at infertility treatment. Series of women who became pregnant after cabergoline treatment showed no increased rate of birth defects [62,63,64,65]. A total of 491 infants were born. According to the publications, 30 had a congenital anomaly but only 16 seemed to have real congenital malformations, 3%, which is probably a normal rate. Very little epidemiological data exist on the influence of maternal use of drugs for Parkinson’s disease and neonatal outcome [66].

**Table 16:** Number of infants exposed in early pregnancy for drugs used for Parkinson’s disease.

ATC	Drug name	Early exposures	Late exposures
N04AA01	trihexyphenidyl	7	8
N04AA02	biperiden	14	16
N04AA03	metixene	7	-
N04AB02	orphenadrine	26	-
N04BA02/03	levodopa+decarboxylase inhibitor	27	10
N04BB01	amantadine	2	-
N04BC01	bromocriptin	20	-
N04BC04	ropinirol	3	1
N04BC05	pramiperol	31	30
N04BC06	kabergolin	29	2
N04BC07	amomorphine	1	-
N04BD01	selegiline	4	-
N04BX02	entacapone	-	1

#### 3.3.2. Data from the Swedish Medical Register

Table 16 shows the exposures for drugs used for treatment of Parkinson’s disease during pregnancy. Only 145 women reported such drug use. Among the infants born only five had a congenital malformation, four of them were classified as “relatively severe” (RR = 0.67, 95% CI 0.18–1.71). Two of these children had cardiac septum defects, two had hypospadias. In data from the Medical Birth Register, there were only 68 women with 72 infants born. The power of the study is therefore low. There were five preterm singletons (expected number 4.6), five with a low birth weight (3.3 expected), and 12 with neonatal morbidity (9.1 expected, RR = 1.35, 95% CI 0.72–2.53). Six of these infants had respiratory problems (3.9 expected, RR = 1.32 (95% CI 0.57–2.59).

Treatment of Parkinsonism during pregnancy is rare and neither in the literature, nor in the present data set have enough many cases been identified to permit an evaluation of possible risks with such drugs. So far nothing has appeared which could indicate a risk but more information is needed.

### 3.4. Neuroleptics (antipsychotics)

#### 3.4.1. Literature Review

Gentile [67] published a review of antipsychotic therapy during pregnancy. Many of the reports concerned single or only a few exposures.

**Table 17:** Summary of literature on neuroleptics according to the review by Gentile [67].

Drug	Number exposed	Number malformed
Phenothiazines as a group	3,303	68 *
Chlorpromazine	260	4
Prochlorperazine	300	14
Trifluoperazine	539	8
Fluphenazine	261	6
Thioridazine	56	1
Thioethylperazine	33	1
Promethazine	163	7
Perphenazine	90	2
Levopromazine	50	2
Haloperidol	403	10
Pimozide	5	0
Penfluridol	27	1
Flupenthixol	98	3
Chlorprothixen	5	0
Zuclopenthixol	75	9
Clozapine	202	20
Olanzapine	463	26
Quetiapine	214	8
Risperdone	313	14

* In the Gentile paper there is a mistake stating 153 malformed infants among 315 – should be 11, quoted from Romeau-Rouquette *et al*.

**Figure 3:**
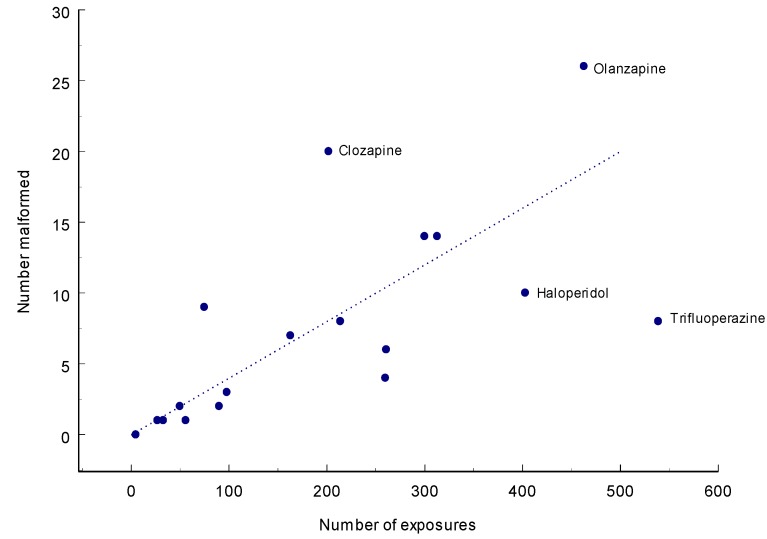
Number of exposures and number of malformations for various antipsychotics according to Gentile [67].

Reports containing at least five exposed infants and with exposures during the first trimester are summarized in Table 17 and data are shown diagrammatically in Figure 3. It can be seen that the average rate of malformed infants was 20/500, 4% which seems high but as no clear definition of malformations has been used it is difficult to state the population rate. Two drugs seem to lie higher than the other, clozapine and olanzapine - the latter may well be random. Two are low: haloperidol and trifluoperazine. The former may be random, the latter material consists mainly of very early (1963) data in a Letter from Smith, Kline and French [68]. The main use (87%) of trifluoperazine was at NVP and not as an antipsychotic.

Since the Gentile review [67], an analysis was published of the effect of mode stabilizers at bipolar disorder [69]. One reported 113 exposed infants in early pregnancy with four malformations. Exposure was determined from a prescription register and it is not certain that women who were supposed to have used the drugs actually had done so. A retrospective case-control study found an association between maternal use of ondansetron and infant cleft palate [70].

In the review by Gentile [67] both first (FGA) and second (SGA) generation antipsychotics were associated with neonatal complications including withdrawal symptoms, extrapyramidal signs and respiratory problems. SGA seemed to increase the risk for gestational diabetes resulting in large for gestational age infants. A nearly doubled risk of gestational diabetes after maternal use of antipsychotics was observed without finding a significant difference between olanzapine or clozapine (SGA) and other neuroleptics [71]. Use of SGA during pregnancy may increase the risk for excessive weight gain, increased serum triglyceride and cholesterol level, glucose intolerance and gestational diabetes.

To this drug group belongs *lithium*. The story about lithium and heart defects started in 1976 with the analysis of infants born after maternal use of lithium during pregnancy, collected in a register [72]. Among 225 exposed infants reported in 1983, 25 had major congenital malformations and 18 had heart defects, six of these were Ebstein’s anomaly. The early results were discussed by Warkany [73] and it was concluded that the teratogenic effect of lithium was not verified. As a comment to this, an international study was reported of 25 Ebstein’s anomaly and 44 other tricuspidal malformations and age-parity matched controls—none of the cases were exposed to lithium, and the same was true for 15 further cases which lacked controls [74]. A case-control study of lithium and Ebstein’s anomaly [75] used children with neuroblastoma as controls. Among 59 cases with Ebstein’s anomaly, no mother had used lithium, among the 168 neuroblastoma controls there was one such mother.

A prospective study [76] of 105 lithium-exposed infants found a congenital defect only in three, one with Ebstein’s anomaly and one with a myelomeningocele (but in the latter case the woman had also used carbamazepine which seems to increase the risk for a neural tube defect).

A review on the use of lithium during pregnancy and neonatal outcome summarized the available case-control studies and found a total of 222 Ebstein cases and 518 controls—only one lithium exposure was identified, a control [77]. The authors conclude that lithium may add a small risk for a congenital heart malformation. Another review [78] stated that the risk of teratogenesis after maternal use of lithium was lower than previously thought.

Even though lithium exposure sometimes may exist associated with Ebstein’s anomaly [79], it does not seem to be an important reason for this rare malformation (approximate prevalence 1/20,000 births). An association with other cardiac defects may exist but is not definitely proved.

Maternal use of lithium during pregnancy may affect neonatal conditions of the infant. This was summarized by Gentile [79]. Prematurity combined with large-for-gestational age has been described and also neonatal morbidity as cyanosis, flaccidity and cardiac arrhythmias. Cases have also been described of nephrogenic diabetes insipidus and hypothyroidism. No special studies of the effect of maternal use of dixyrazine or prochlorperazine on neonatal morbidity seem to exist.

#### 3.4.2. Data from the Swedish Medical Birth Register

Table 18 summarizes neuroleptics reported in early or late pregnancy. Two drugs dominate: dixyrazine and prochlorperazine. These drugs are mainly used for nausea and vomiting in pregnancy and will therefore be treated separately. Early exposure for other neuroleptic drugs was relatively rare. The main use of dixyrazine and prochlorperazine is in early pregnancy and only 126 women with 128 infants had got prescriptions after the first trimester. Other neuroleptics had been prescribed to 584 women who had 592 infants.

**Table 18:** Number of infants exposed in early pregnancy for neuroleptics or born by women who had filled prescriptions for neuroleptics during the 2nd or 3rd trimester.

ATC	Drug name	FGA/SGA	Early exposure	Late Exposure
N05AA01	chlorpromazine	FGA	10	-
N05AA02	levomepromazine	FGA	89	39
N05AB01	dixyrazine	-	2,597	109
N05AB02	fluphenazine	FGA	16	1
N05AB03	perphenazine	FGA	117	27
N05AB04	prochlorperazine	-	315	20
N05AC02	thioridazine	FGA	33	-
N05AD01	haloperidol	FGA	115	36
N05AD03	melperone	SGA	3	-
N05AE04	ziprasidone	SGA	5	9
N05AF01	flupentixzol	FGA	154	32
N05AF03	chlorprotixene	FGA	10	6
N05AF05	zuclopenthixcol	FGA	95	22
N05AG02	pimozide	FGA	6	-
N05AH02	clozapine	SGA	32	9
N05AH03	olanzapine	SGA	205	101
N05AH04	quetiapine	SGA	75	57
N05AN01	lithium	-	234	122
N05AX08	risperidone	SGA	93	49
N05AX12	aripiprazol	SGA	32	30
N05AX13	paliperidone	SGA	1	-
N05A	unspecified	-	19	-

FGA = first generation antipsychotics; SGA = second generation antipsychotics.

**Figure 4:**
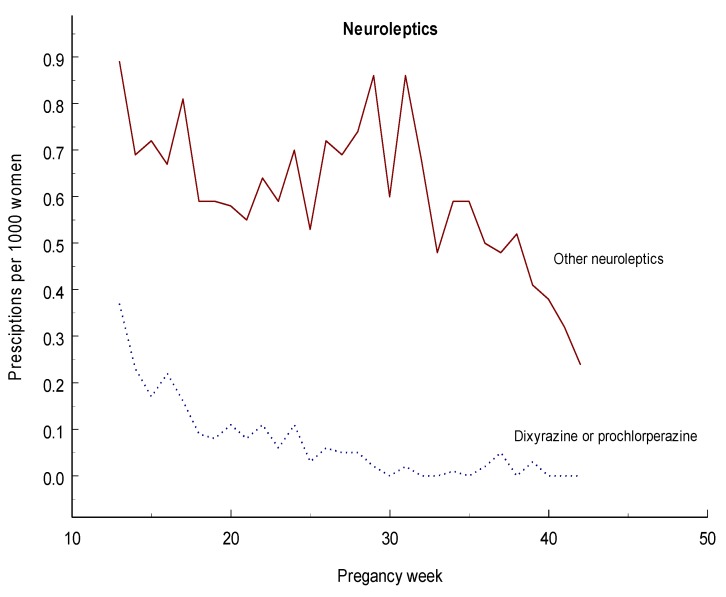
Diagram showing number of women per 1,000 who had filled a prescription for a neuroleptic for each gestational week during the second or third trimester.

Figure 4 shows that the prescription of dixyrazine or prochlorperazine mainly occurred in early 2nd trimester while the prescription of the other neuroleptics declined moderately during pregnancy.

Table 19 shows that there was a generally increased risk for malformations after the use of neuroleptics but no specific malformation showed a significant over-risk but the estimated RRs were high for pyloric stenosis, pes equinovarus, and hypospadias but confidence intervals were wide.

**Table 19:** Congenital malformations in infants exposed in early pregnancy for neuroleptics other than dixyrazine or prochloperazine. Bold text marks statistical significance.

Malformation	Number with drug	Total number	OR/RR	95% CI
All	78	70,339	**1.33**	**1.05–1.60**
Relatively severe	60	48,499	**1.48**	**1.13–1.92**
Chromosome anomaly	3	2,932	0.96	0.20–2.82 #
Neural tube defects	1	734	-	-
Other CNS malformations	2	1,131	-	-
Severe eye malformations	0	579	-	-
Severe ear malformations	1	280	-	-
Orofacial clefts	1	2,756	-	-
Any cardiac defect	13	16,145	0.83	0.48–1.41
Septal cardiac defect	9	11,157	0.83	0.44–1.59
Oesophageal atresia	1	445	-	-
Small gut atresia	0	392	-	-
Anal atresia	0	590	-	-
Pyloric stenosis	3	1,101	2.83	0.58–8.27 #
Abdominal wall defect	2	413	-	-
Diaphragmatic hernia	0	368	-	-
Hypospadias	9	4,552	1.77	0.81–3.36 #
Major renal malformations	2	882	-	-
Pes equinovarus	4	2,127	1.98	0.54–5.07 #
Poly- or syndactyly	4	3,084	0.85	0.23–2.18 #
Limb reduction defects	1	830	-	-
Craniostenosis	0	862	-	-

# RR from observed/expected numbers with exact 95% CI based on Poisson distributions.

When specific drugs with at least 100 exposures were studied (Table 20), the only statistically just significant effect was seen with flupentizol. Among the 12 cases five were urogenital: one infant had renal dysplasia and another polycystic kidney, one had ureter stenosis and another had hydronephrosis, and the fifth had hypospadias.

**Table 20:** “Relatively severe malformations” after maternal use of five neuroleptics with at least 100 exposures. Bold text marks statistical significance.

Malformation	Drug	Number Malformed	Total number	OR/RR	95% CI
Relatively severe	perphenazine	2	117	-	-
	haloperidol	6	115	1.21	0.39–2.83 #
	flupentizol	12	154	**1.94**	**1.00–3.40 #**
	olanzapine	6	205	0.93	0.40–1.84 #
	lithium	10	234	1.09	0.52–2.00 #

# RR from observed/expected numbers with exact 95% CI based on Poisson distributions.

Among the ten malformed infants exposed to lithium, three had cardiac defects: one infant had a VSD, one term infant had PDA, and the third had tricuspidal and mitral malformations.

Table 21 shows that after maternal use of dixyrazine or prochlorperazine, the risk for a congenital malformation was actually significantly reduced. This if often seen with drugs used for nausea and vomiting in pregnancy which is thought to be associated with a well-functioning placenta and therefore a better than expected delivery outcome.

**Table 21:** Congenital malformations observed after maternal use of dixyrazine or prochlorperazine. Bold text marks statistical significance.

Malformation	Number with drug	Total number	OR/RR	95% CI
All	121	70,339	**0.82**	**0.68–0.98**
Relatively severe	81	48,499	**0.78**	**0.63–0.98**
Chromosome anomaly	8	2,932	1.27	0.55–2.51
Neural tube defects	0	734	-	-
Other CNS malformations	0	1,131	-	-
Severe eye malformations	0	579	-	-
Severe ear malformations	0	280	-	-
Orofacial clefts	3	2,756	1.70	0.05–4.98 #
Any cardiac defect	29	16,145	0.83	0.58–1.20
Septal cardiac defect	23	11,157	0.97	0.65–1.47
Oesophageal atresia	1	445	-	-
Small gut atresia	1	392	-	-
Anal atresia	0	590	-	-
Pyloric stenosis	2	1,101	-	-
Abdominal wall defect	2	413	-	-
Diaphragmatic hernia	2	368	-	-
Hypospadias	9	4,552	0.98	0.45–1.86
Major renal malformations	1	882	-	-
Pes equinovarus	0	2,127	-	-
Poly- or syndactyly	10	3,084	1.50	0.72–2.76
Limb reduction defects	0	830	-	-
Craniostenosis	1	862	-	-

# RR from observed/expected numbers with exact 95% CI based on Poisson distributions.

The only outcome which was significantly affected by the use of dixyrazine or prochlorperazine during the 2nd or 3rd trimester was placental abruption but this was based on only three cases (RR = 6.67, 95% CI 1.37–19.5) and may be random in spite of formal statistical significance.

Table 22 shows the outcomes after the use of the other neuroleptics/antipsychotics. The size of the material was restricted but an excess was seen of preeclampsia and of hemorrhagic around delivery but neither reached statistical significance. There is a significant excess both of SGA and LGA infants and also of neonatal morbidity, observable both for respiratory and for CNS problems and for low Apgar score.

**Table 22:** Outcomes after maternal use of neuroleptics with the exception of dixyrazine and prochloperazine during the 2nd or 3rd trimester. Bold text marks statistical significance.

Outcome	With neuroleptics	In population	OR	95% CI
Preeclampsia	28	25,688	1.25	0.84–1.86
Gestational diabetes	7	7,118	0.90	0.30–1.86 #
Abruption of placenta	1	2,379	-	-
Haemorrhage around delivery	44	46,981	1.31	0.96–1.79
Induction of delivery	108	81,793	**1.72**	**1.39–2.14**
Preterm birth < 37 weeks	27	31,394	1.02	0.69–1.51
Low birth weight < 2500 g	25	20,712	1.25	0.82–1.91
Small for gestational age	24	16,440	**1.72**	**1.13–2.95**
Large for gestational age	30	19,745	**2.03**	**1.39–2.95**
Neonatal diagnosis	72	67,057	**1.34**	**1.04–1.73**
Respiratory diagnosis	38	27,708	**1.73**	**1.24–2.40**
Hypoglycaemia	16	14,455	1.30	0.79–2.16
Jaundice	18	26,928	0.80	0.50–1.29
CNS diagnoses	13	6,940	**2.18**	**1.16–3.73 #**
Low 5 min Apgar score	19	13,211	**1.72**	**1.09–2.71**

# RR from observed/expected numbers with exact 95% CI based on Poisson distributions.

Most of the infants exposed to antipsychotics were exposed to second generation antipsychotics (258 against 151 exposed to first generation antipsychotics). The risk for preterm birth was lower after first generation antipsychotics (RR = 0.59, 95% CI 0.19–1.37) than after second generation antipsychotics (OR =1.31, 95% CI 0.73–1.99) but this difference may well be random. There was no difference in the rate of neonatal morbidity or specifically in CNS diagnoses after first and second generation antipsychotics.

For individual neuroleptics the risk estimate for neonatal morbidity varied. The highest risks were seen for lithium (OR = 1.91, 95% CI 1.22–3.0) and quetiapine (RR = 1.98, 95% CI 1.04–1.98, based on 11 cases), both higher than olanzapine with OR = 1.19 (95% CI 0.77–1.84). If all antipsychotics are studied, excluding women who also got lithium, the OR = 1.32 (95% CI 1.03–1.69).

There are not enough data available to finally evaluate the possible teratogenic risk of neuroleptics/antipsychotics, but no firm evidence exists of teratogenicity. It is probably wise to try to avoid exposure to these drugs during early pregnancy but if exposure has occurred, there seems to be no major risk which would motivate an interruption of the pregnancy. The use of dixyrazine or prochlorperazine at NVP seems to be without teratogenic risks but too little is known about the use of these drugs as antipsychotics. The teratogenic effect of lithium is unclear but a possible association with an increased risk for cardiovascular defects remains and if possible exposure should be avoided.

After maternal use of antipsychotics during the 2nd or 3rd trimester there was some signs of intrauterine growth disturbances leading to both an increased risk for SGA and for LGA. Neonatal morbidity was increased and specifically for respiratory problems and for CNS diagnoses. The material is not large enough to investigate possible differences between first and second generation antipsychotics. The highest risk estimate was for lithium. – Exposure to dixyrazine or prochlorperazine, mainly during the 2nd trimester and probably mainly because of nausea and vomiting in pregnancy had no measurable effects on the outcomes studied.

### 3.5. Sedatives and Hypnotics

#### 3.5.1. Literature Review

The most well known teratogenic drug, thalidomide, belongs to this drug group which may have contributed to the interest for possible risks associated with the use of such drugs during pregnancy. Notably in the early literature after thalidomide, examples of coincidences between maternal use of a sedative or hypnotic and infant malformations were reported.

The barbiturate group of sedatives/hypnotics was in some early studies linked to an increased risk for congenital malformations (e.g., [80,81]) while in other studies no such link was found (e.g., [15]). In Sweden phenobarbital is nowadays used mainly as an anticonvulsant (see above) and barbiturates used as sedatives/hypnotics have been replaced with other drugs, notably benzodiazepines, hypnotic benzodiazepine receptor agonists (HBRA), hydroxyzine and propiomazine.

Benzodiazepines have also been looked upon as tentative teratogens (e.g., [4,82]) and some authors [83] described a dysmorphic pattern which in a case-control study correlated with maternal blood level of benzodiazepine in week 12. This has not been confirmed and may have been related to abuse situations. A detailed discussion of the early literature was made by Weber [84].

Most large studies have found no evidence for a major teratogenic property of benzodiazepines [85]. One study found an increased risk for any relatively serious malformation after benzodiazepine use but after exclusion of women who had also used anticonvulsants, the risk declined and lost statistical significance [86]. No increase in orofacial clefts was seen – the only specifically increased risk was for alimentary tract atresia (oesophageal or anal) after the use of benzodiazepine or HBRA with seven cases against 2.6 expected, and pyloric stenosis with seven cases against 3.8 expected. Among the 14 cases all but one had been exposed to benzodiazepines and two to HBRA (one case had both diazepam and zolpidem). One other report in the literature has linked use of benzodiazepines with an increased risk for alimentary atresia: anal atresia and lorazepam [87].

HBRA drugs have been studied less often. Except for a number of small cohort studies only the above-mentioned study [86] with a follow-up study [88] exists. The latter study comprised 1 341 infants whose mothers had reported the use of HBRA in early pregnancy. The risk for any relatively severe malformation was 0.95 (95% CI 0.69–1.30) and the only abnormality in outcome was five infants with alimentary tract malformations other than atresias: two gut duplications, one Meckel diverticulum, one unspecified gut malformation, and one megacolon. This was most likely a random finding even though formal statistical significance was reached. 

Propiomazine use during pregnancy has been very little studied. The largest study comprised 1 086 infants exposed in early pregnancy with 37 malformed individuals, OR = 1.05 (95% CI 0.75–1.47) [8].

Maternal use of benzodiazepines during late pregnancy has been associated with neonatal morbidity [89], including withdrawal symptoms [90] and the so-called “floppy baby” syndrome [91]. An increased risk for preterm birth, low birth weight, and small-for-gestational age is also known but if this is due to drug use or underlying pathology can be debated. NAS symptoms may occur as a result of benzodiazepine treatment towards the end of the pregnancy [92]. An increased risk for preterm birth and low birth weight was found in singleton infants exposed to benzodiazepines or HBRA during late pregnancy and also an increased rate of infants with a low Apgar score, also among term infants [85]. An increased risk for respiratory problems was also seen.

**Table 23:** Number of infants exposed in early pregnancy or during the 2nd or 3rd trimester for sedatives or hypnotics.

ATC	Drug name	Early exposure	Late exposure	ATC	Drug name	Early exposure	Late exposure
N05BA01	diazepam	865	492	N05CD03	flunitrazepam	184	127
N05BA02	bromazepam	3	-	N05CD05	triazolam	97	169
N05BA04	oxazepam	844	1,066	N05CD08	midazolam	18	2
N05BA06	lorazepam	35	16	N05CF01	zopilcone	1,143	897
N05BA08	bromazepam	2	-	N05CF02	zolpidem	483	990
N05BA09	clobazam	24	4	N05CF03	zaleplon	29	39
N05BA12	alprazolam	444	248	N05CH01	melatonin	24	20
N05BB01	hydroxyzine	1,190	1,141	N05CM02	clomethiazole	3	5
N05BC01	meprobamate	1	-	N05CM05	scopolamine	1	-
N05BE01	buspirone	175	26	N05CM06	propiomazine	1,953	2,203
N05CA01	pentobarbital	3	-	N05CM09	valerian	129	17
N05CB02	barbiturate, combination	7	-	N05CP01	baldrian root	3	-
N05CC01	chloral hydrate	1	-	N05B	unpec.sedative	21	-
N05CD02	nitrazepam	134	169	N05C	uspec.hypnotic	38	-

#### 3.5.2. Data from the Swedish Medical Birth Register

**Figure 5:**
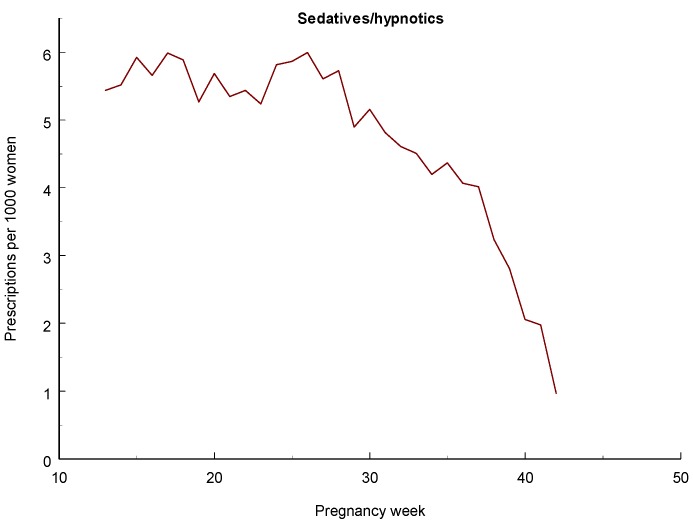
Diagram showing number of women per 1,000 who had filled a prescription for a sedative or hypnotic drug for each gestational week.

The use of sedatives or hypnotics in early and late pregnancy is shown in Table 23. Prescriptions for sedatives or hypnotics were filled by 5,545 women with 5,665 infants. Benzodiazepines had been prescribed to 1,890 women with 1,932 infants, hypnotic benzodiazepine receptor agonists (HBRA) to1 778 women with 1,814 infants. Among other sedatives/hypnotics two drugs had been prescribed to many women: hydroxyzine to 1,117 women with 1,141 infants and propiomazine to 2,161 women with 2,203 infants. Figure 5 shows that prescription of sedatives/hypnotics markedly declines during pregnancy progress.

Infants with congenital malformations are summarized in Table 24. No significant associations were seen between use of sedatives/hypnotics and any malformation or specific groups of malformations. The RR estimates were increased for small gut and anal atresia and for pyloric stenosis, but if all types of alimentary tract atresia/stenosis were analyzed together, the increased OR was still not significant (1.32, 95% CI 0.87–2.00).

**Table 24:** Congenital malformations in infants whose mothers had used sedatives or hypnotics in early pregnancy.

Malformation	Number with drug	Total number	OR/RR	95% CI
All	358	70,339	0.91	0.82–1.01
Relatively severe	272	48,499	1.00	0.81–1.12
Chromosome anomaly	21	2,932	1.07	0.69–1.66
Neural tube defects	0	734	-	-
Other CNS malformations	4	1,131	0.72	0.20–1.84 #
Severe eye malformations	3	579	1.05	0.22–3.08 #
Severe ear malformations	2	280	-	-
Orofacial clefts	11	2,756	0.70	0.39–1.26
Any cardiac defect	75	16,145	0.82	0.61–1.03
Septal cardiac defect	56	11,157	0.87	0.67–1.13
Oesophageal atresia	1	445	-	-
Small gut atresia	4	392	1.62	0.44–4.15 #
Anal atresia	4	590	1.19	0.33–3.06 #
Pyloric stenosis	13	1,101	1.81	0.96–3.09 #
Abdominal wall defect	1	413	-	-
Diaphragmatic hernia	1	368	-	-
Hypospadias	29	4,552	1.20	0.84–1.73
Major renal malformations	1	882	-	-
Pes equinovarus	11	2,127	0.77	0.43–1.37
Poly– or syndactyly	18	3,084	1.05	0.67–1.66
Limb reduction defects	6	830	1.16	0.42–2.52 #
Craniostenosis	4	862	0.93	0.25–2.38

# RR from observed/expected numbers with exact 95% CI based on Poisson distributions.

When associations were studied between subgroups of sedatives/hypnotics and specific malformations, some statistical significances appeared (Table 25). There was a nearly doubled risk of any “relatively severe malformation” after use of alprazolam and this was mainly due to an association with cardiovascular defects. All ten cases had septum defects: six had VSD, two had ASD and two both VSD and ASD. One of the infants with a VSD also had CoA and one with ASD also had a tricuspidal malformation.

**Table 25:** Congenital malformations in infants whose mothers had used some specific sedatives or hypnotics. Bold text marks statistical significance.

Malformation	Drug	Number with drug	Total number	OR	95% CI
Relatively severe					
	all benzodiazepines	108	2,537	1.11	0.92–1.35
	diazepam	32	865	0.94	0.67–1.33
	oxazepam	33	843	1.01	0.72–1.41
	alprazolam	31	444	**1.97**	**1.38–2.02**
	nitrazepam	1	134	-	-
	flunitrazepam	8	184	1.02	0.44–2.01#
	hydroxyzine	42	1,190	0.93	0.79–1.18
	propiomazine	72	1,953	0.97	0.79–1.18
	all HBRA	40	1,642	0.77	0.61–0.98
	zopiclone	36	1,143	0.87	0.62–1.21
	zolpidem	12	403	0.81	0.46–1.42
Any cardiac defects					
	all benzodiazepines	40	2,537	1.25	0.92–1.71
	diazepam	11	865	1.03	0.57–1.85
	oxazepam	11	843	0.99	0.55–1.77
	alprozalam	13	444	**2.43**	**1.42–4.15**
	nitrazepam	0	134	-	-
	flunitrazepam	3	184	1.20	0.25–3.51#
	hydroxyzine	9	1,190	0.62	0.33–1.20
	propiomazine	21	1,953	0.79	0.53–1.17
	all HBRA	11	1,642	0.54	0.32–0.91
	zoplicone	9	1,143	0.67	0.35–1.29
	zolpidem	2	403	0.30	0.04–1.07#
Orofacial clefts	all benzodiazepines	3	2,537	0.54	0.11–1.59#
	hydroxine	3	1,190	1.28	0.26–3.73#
	propiomazine	1	1,953	-	-
	all HBRA	3	1,642	0.75	0.15–2.19#
Pyloric stenosis	all benzodiazepines	8	2,537	**3.31**	**1.43–6.51#**
	hydroxyzine	0	1,190	-	-
	propiomazine	2	1,953	-	-
	all HBRA	4	1,642	0.70	0.19–1.79#
Pes equinovarus	all benzodiazepines	5	2,537	0.96	0.31–2.24#
	hydroxyzine	2	1,190	0.88	0.11–3.20#
	propiomazine	5	1,952	1.36	0.49–3.17#
	all HBRA	5	1,642	1.19	0.09–2.77#
Poly– or syndactyly	all benzodiazepines	4	2,537	0.65	0.18–1.67#
	hydroxyzine	2	1,190	0.75	0.09–2.72#
	propiomazine	8	1,952	1.53	0.66–3.02#
	all HBRA	5	1,642	1.19	0.39–2.77#

# RR from observed/expected numbers with exact 95% CI based on Poisson distributions.

Another formally statistically significant association was between benzodiazepines and pyloric stenosis. Among the eight infants with this combination, three had used alprazolam, two diazepam, two flunitrazepam, and one oxazepam. There was no association between other sedatives/hypnotics than benzodiazepines and pyloric stenosis.

**Table 26:** Outcomes after maternal use of sedatives or hypnotics during the 2nd or 3rd trimester. Bold text marks statistical significance.

Outcome	With sedatives or hypnotics	In Population	OR	95% CI
Preeclampsia	283	25,688	**1.26**	**1.11–1.42**
Gestational diabetes	91	7,118	1.23	0.99–1.52
Abruption of placenta	32	2,379	1.33	0.93–1.89
Haemorrhage around delivery	456	46,981	**1.13**	**1.03–1.25**
Induction of delivery	1,087	81,793	**1.59**	**1.48–1.70**
Preterm birth < 32 weeks	60	4,881	1.24	0.96–1.61
Preterm birth < 37 weeks	389	31,394	**1.36**	**1.22–1.51**
Low birth weight < 2500 g	261	20,712	**1.31**	**1.15–1.49**
Small for gestational age	187	16,440	**1.18**	**1.02–1.37**
Large for gestational age	199	19,745	1.12	0.97–1.30
Neonatal diagnosis	894	67,057	**1.53**	**1.42–1.64**
Respiratory diagnosis	395	27,708	**1.60**	**1.44–1.78**
Hypoglycaemia	194	14,455	**1.38**	**1.19–1.60**
Jaundice	262	26,928	1.02	0.90–1.10
CNS diagnoses	96	6,940	**1.58**	**1.28–1.93**
Low 5 min Apgar score	257	13,211	**2.18**	**1.93–2.48**

Outcomes after use of any sedative or hypnotic during the 2nd or 3rd trimester are shown in Table 26. The risk of preeclampsia is significantly increased while the risks for gestational diabetes and for abruption of placenta are similarly increased but statistical significance is not quite reached. Haemorrhage around delivery is significantly increased.

There is a significantly increased risk of preterm birth, low birth weight and SGA. The risk for neonatal morbidity is increased and this is seen also for respiratory diagnoses, CNS diagnoses and low 5 min Apgar score. Table 27 compares the risk estimates for the above mentioned four groups of sedatives/hypnotics. They differ somewhat but in most instances this can be result of random fluctuations around a common risk estimate. There is a tendency that the effect on preterm birth, low birth weight and neonatal diagnoses are higher and similar for benzodiazepines and HBRA than for the other two drugs. A significantly increased risk for LGA was seen only for propiomazine. 

**Table 27:** Neonatal outcome for four different groups of sedatives/hypnotics: benzodiazepine, HBRA, hydroxyzine, and propiomazine. Bold text marks statistical significance.

	Benzodiazepines			HBRA			Hydroxyzine			Propriomazine		
Outcome	No.	OR	95% CI	No.	OR	95% CI	No.	OR	95% CI	No.	OR	95%CI
<37 weeks	157	**1.62**	**1.37–1.91**	150	**1.62**	**1.37–1.92**	76	1.26	0.99–1.60	130	1.14	0.96–1.37
<2500 g	97	**1.40**	**1.14–1.72**	103	**1.62**	**1.33–1.97**	48	1.13	0.84–1.52	98	**1.28**	**1.04–1.57**
SGA	58	1.23	0.96–1.58	68	**1.37**	**1.07–1.75**	34	1.02	0.72–1.44	79	**1.30**	**1.04–1.64**
LGA	72	1.17	0.92–1.48	60	1.04	0.80–1.35	36	1.01	0.72–1.41	99	**1.46**	**1.19–1.79**
Neonatal diagnosis	342	**1.74**	**1.55–1.96**	310	**1.64**	**1.45–1.85**	161	**1.32**	**1.12–1.56**	342	**1.50**	**1.33–1.68**
Respiratorydiagnosis	157	**1.60**	**1.44–1.78**	144	**1.80**	**1.52–2.23**	70	**1.57**	**1.21–1.98**	146	**1.52**	**1.28–1.80**
Hypo–glycaemia	194	**1.38**	**1.19–1.60**	144	**1.80**	**1.12–2.13**	40	**1.42**	**1.03–1.96**	81	**1.49**	**1.19–1.86**
Jaundice	99	1.21	0.98–1.48	86	1.10	0.88–1.36	55	1.07	0.82–1.41	102	1.10	0.90–1.35
CNS diagnosis	96	**1.58**	**1.18–1.93**	40	**2.00**	**1.46–2.73**	11	0.95	0.52–1.72	35	**1.44**	**1.07–2.01**
Low Apgar score	257	**2.18**	**1.93–2.48**	105	**2.78**	**2.29–2.36**	37	**1.56**	**1.11–2.19**	92	**1.96**	**1.59–2.41**

There seems to exist no obvious teratogenicity of benzodiazepines and other sedatives/hypnotics. There is a suggested effet of alprazolam which has to be investigated in an independent material and a possible relationship between benzodiazepines and pyloric stenosis but both observations were based on rather few exposures.

There are clear-cut associations between 2^nd^ or 3^rd^ trimester maternal use of sedatives or hypnotics and many outcome variables. There is a tendency that these are stronger for benzodiazepines and HBRA than for the other two commonly used hypnotics. It is difficult to decide if these are drug effects or if these drugs are prescribed to women with complicated pregnancies, resulting in preterm birth etc. The suggested differences between benzodiazepines or HBRA and other hypnotics may suggest some drug specificity, indicating a direct drug effect. This may be most likely for effects on neonatal CNS diagnoses. When possible, the use of sedatives and hypnotics should be kept low during the late part of the pregnancy. It should also be stressed that the methodology used may not identify less specific effects on the neonates, not identifiable from ICD codes.

### 3.6. Antidepressants

#### 3.6.1. Literature Review

A very large literature exists on the use of antidepressants and notably SSRI drugs during pregnancy and the presence of congenital malformations. There are three main classes of antidepressants which will be treated separately: tricyclic antidepressants (TCA), selective serotonin reuptake inhibitors (SSRI), and serotonin/noradrenalin inhibitors (SNRI/NRI).

*TCA*. Relatively little has been published on this group of antidepressants. There were some early studies which suggested a teratogenic effect of these drugs [3] but most likely these observations were random events. Recently, studies have linked use of TCA and notably clomipramine with an increased risk for congenital malformations and especially cardiovascular defects [93]. The risk estimate for any major congenital malformation was 1.36 (95% CI 1.07–1.72), for any cardiovascular defect 1.63 (95% CI 1.12–2.36), and for septum defects 1.84 (95%CI 1.13–2.97). The explanation to this phenomenon was sought in the inhibiting effect of clomipramine on a specific cardiac potassium current channel.

*SSRI*. The studies of the possible teratogenic effects of SSRI can be divided into some different groups. Teratology Information Service (TIS), data from hospital records of health systems, industry pregnancy registers, case-controls studies, prescription registers, and the Swedish Medical Birth Register (the only category with prospective population-based interviews in early pregnancy) (Table 28).

**Table 28:** Summary of literature on the association between antidepressant use and infant congenital malformations. Numbers of exposed infants within brackets.

Source and authors	Year	Drugs and number of women
*TIS data*		
Pastuszak *et al*. [94]	1993	fluoxetine (128)
Chambers *et al*. [95]	1996	fluoxetine (228)
McElhatton *et al*. [96]	1996	fluoxetine (96), fluvoxamine (66), paroxetine (3)
Kulin *et al*. [97]	1998	fluvoxamine, paroxetine, sertraline (267)
Sivojelezova *et al*. [98].	2005	citalopram (125)
Einarsson *et al*. [99]	2008	paroxetine (1,174)
Diav-Citrin *et al*. [100]	2008	paroxetine (410), fluoxetine (314)
Einarson *et al*. [101]	2009	bupropion (113), citalopram (184), escitalopram (21), fluvoxamine (52), nefazodone (49), paroxetine (148), mirtazepine (68), fluoxetine (81), trazadone (17), venlafaxine (154), sertraline (61), all antidepressants 928
Einarson *et al*. [102]	2011	2 or 3 antidepressants (89)
*Hospital records and* *Health systems*		
Hendrick *et al*. [103]	2003	SSRI (147)
Cole *et al*. [104]	2007	paroxetine (989)
Davis *et al*. [105]	2007	SSRI (805) among them paroxetine (134)
*Industry pregnancy* * registries*		
Goldstein *et al*. [106]	1997	fluoxetine (759)
Cole *et al*. [107]	2006	bupropion (1,213)
*Case-control studies*		
Simon *et al*. [108]	2002	SSRI (12 major, 18 minor malf.)
Louik *et al*. [109]	2007	SSRI (18 malformation classes)
Alwan *et al*. [110]	2007	SSRI (26 malformation classes)
Bakker *et al*. [111]	2009	paroxetine (cardiovascular defects)
Alwan *et al*. [112]	2010	bupropion (cardiovascular defects)
Polen *et al*. [113]	2013	venlafaxine (24 malformation classes)
*Prescription registers*		
Malm *et al*. [114]	2005	citalopram (554), fluoxetine (525), paroxetine (152), sertraline (118), fluvoxamine (65) – all SSRI 1,398
Wogelius *et al*. [115]	2006	SSRI (1,051)
Bérard *et al*. [116]	2007	paroxetine (443), sertraline (186), citalopram (113), fluoxetine (101), fluvoxamine (43) – all SSRI 985
Oberlander *et al*. [117]	2007	citalopram (101), fluoxetine (638), fluvoxamine (119), paroxetine (993), sertraline (608) – all SSRI 2,734.
Pedersen *et al*. [118]	2009	fluoxetine (348), citalopram (460), paroxetine (299), sertraline (259), multiple SSRI (193) – all SSRI 1,370
Kornum *et al*. [119]	2010	fluoxetine (472), sertraline (352), paroxetine (297), citalopram (658), escitalopram (88) – all SSRI 2,062
Jimenez-Solem *et al*. [120].	2012	SSRI (4183)
*Swedish Medical Birth Register*		
Ericson *et al*. [121]	1999	fluoxetine (16), citalopram (375), paroxetine (131), sertraline (32) – all SSRI 546.
Källén [122]	2004	fluoxetine (512)
Källén & Otterblad Olausson [123]	2007	fluoxetine (860), citalopram (2,579), paroxetine (908), sertraline (1,807), fluvoxamine (36), escitalopram (66) – all SSRI 6,481
Reis & Källén [93]	2010	fluoxetine (1,522), citalopram (3,950), paroxetine (1,208), sertraline (3,297), fluvoxamine (42), escitalopram (153) – all SSRI 10,170

Only the three Danish studies [115,119,120] found statistically significant risk increases for any congenital malformation. The largest Swedish study [93] showed a slight but not significant risk increase (Table 29). The problem with the Danish studies is that they all identified congenital malformations only from hospital discharge diagnoses. Infants who were exposed to antidepressants during pregnancy are more likely than other children to be transferred to neonatal wards because of neonatal morbidity. This may explain a high identification of conditions which otherwise would not have been registered.

**Table 29:** Publications describing risks for any malformation after maternal use of antidepressants. Bold text marks statistical significance.

Author	Year	Country	No. malformed	Risk estimate	Malformations included
*Any malformation*					
Kulin *et al*. [97]	1998	US/Canada	9	1.06 (0.43–2.62)	Major
Malm *et al*. [114]	2005	Finland	75	1.0 (0.6–1.7)	Major
Wogelius *et al*. [115]	2006	Denmark	17	**1.34 (1.00–1.79)**	Some excluded
Davis *et al*. [105]	2007	USA	108	0.97 (0.81–1.16)	“Of interest”
Oberlander *et al*. [117]	2007	Canada	75	Risk diff −0.28 (−0.86–0.43)	Major
Kornum *et al*. [119]	2009	Denmark	105	**1.3 (1.1–1.6)**	Any
Einarson *et al*. [99]	2009	Canada	30	0.9 (0.5–1.61)	Major
Reis & Källén [93]	2010	Sweden	345	1.08 (0.97–1.21)	Rel. severe
Jimenez–Solem *et al*. [120]	2012	Denmark	208	**1.33 (1.16–1.53)**	Major

Most interest has been shown cardiovascular defects, notably after the preliminary results that there may exist a relationship between paroxetine and septal defects [104]. Table 30 includes studies with reasonable numbers of exposed cases (at least 10).

**Table 30:** Publications giving risks for cardiovascular defects after maternal use of antidepressants. Bold text marks statistical significance.

Authors	Year	Drugs	No. of defects	OR/risk difference (=RD)	Note
Cole *et al*. [107]	2006	bupropion	13	NS	compared with other antidepr.
Louik *et al*. [109]	2007	SSRI	100	1.2 (0.9–1.6)	
		fluoxetine	31	0.9 (0.6–1.5)	
		paroxetine	25	1.4 (0.8–1.25)	
		sertraline	32	1.5 (0.9–2.6)	
Cole *et al*. [104]	2007	paroxetine	17	1.68 (0.95–2.97)	compared with other antidepr.
Oberlander *et al*. [117]	2008	SSRI	17	RD 0.21(−0.14–0.56)	
Reis and Källén [93]	2010	SSRI	109	1.08 (0.97–1.21)	
		fluoxetine	21	1.31 (0.85–2.02)	significant
		citalopram	37	0.86 (0.62–1.20)	difference
		paroxetine	24	**1.66 (1.09–2.53)**	between
		sertraline	26	0.74 (0.50–1.09)	drugs
Bakker *et al*. [111]	2010	paroxetine	10	1.5 (0.5–4.0)	
Kornum *et al*. [119]	2010	SSRI	26	**1.7 (1.1–2.5)**	
Jimenez–Solem *et al*. [120]	2012	SSRI	77	**2.01 (1.60–2.53)**	
		Low dose	44	**1.83 (1.35–2.48)**	
		High dose	33	**2.26 (1.60–3.19)**	

The largest materials were thus that by Louik *et al*. [109] and that by Reis and Källén [93]. The former is a case-control study with risk for recall bias and a considerable non-response rate. In the Reis and Källén study, a significant risk increase was only seen for paroxetine but the estimate was increased also for fluoxetine, although not statistically significant. A not quite significantly increased risk for any major malformation after fluoxetine (OR = 1.7, 95% CI 0.9–3.3) has been described in a Finnish study [114]. A large part of cardiovascular malformations are made up of septal defects, ventricular (VSD) or atrial (ASD) septum defects (Table 31). 

**Table 31:** Publications giving risk for cardiac septum defects after maternal use of antidepressants. Bold text marks statistical significance.

Author	Year	Drugs	No of defects	OR	Note
Louik *et al*. [109]	2007	SSRI	32	1.2 (0.8–1.8)	VSD, ASD *etc*.
		fluoxetine	10	1.0 (0.5–2.2)	
		sertraline	13	**2.0 (1.2–4.0)**	
Alwan *et al*. [112]	2007	SSRI	43	1.2 (0.7–2.9)	VSD, ASD
Pedersen *et al*. [118]	2009	SSRI	12	**1.99 (1.13–3.53)**	
Reis and Källén [93]	2010	SSRI	61	1.00 (0.77–1.29)	VSD, ASD
		paroxetine	24	1.61 (0.83–2.82)	
Kornum *et al*. [119]	2010	SSRI	18	1.4 (0.8–2.3)	All septal defect types
Jeminiz-Solem *et al*. [120]	2012	SSRI	49	**2.04 (1.52–2.72)**	
Polen *et al*. [113]	2013	venlafaxine	18	**3.0 (1.4–6.4)**	VSD, ASD

Non-cardiovascular malformations have been studied in some studies (Table 32). The studies of Louik *et al*. [109], Alwan *et al*. [110] and Polen *et al*. [113] are all retrospective with a risk of recall bias. The only association which seems possibly true is with hypospadias but also this could be a result of multiple testing. On the other hand, numbers of specific malformations are usually rather low and the confidence interval wide why a true effect can have been hidden.

**Table 32:** Publications with information on other congenital malformations than cardiovacular defects after maternal use of antidepressants. Numbers within [] show the number of exposed specific malformations. Bold text marks statistical significance.

Malformation	Louik *et al*. Reference [109]	Alwan *et al*. Reference [110]	Reis and Källén Reference [93]	Polen *et al*. Reference [113]
Neural tube defect	[5]	-	[2]	-
	0.6 (0.2–1.4)		0.45 (0.05–1.62)	
anencephaly	-	[9]	-	[4]
		**2.4 (1.1–5.1)**		**6.3 (1.5–20.2)**
spina bifida	-	[7]	[2]	[3]
		0.7 (0.3–1.7)		2.1 (0.4–7.6)
Orofacial clefts	-	-	[23]	-
			0.99 (0.66–1.50)	
cleft lip	[22]	[22]	-	[6]
	1.5 (0.9–2.5)	1.0 (0.6–1.6)		1.5 (0.5–4.3)
median cleft palate	[7]	[11]	-	[7]
	0.9 (0.4–2.0)	0.9 (0.4–1.7)		**3.3 (1.1–8.8)**
Abdominal wall defects	-	-	[7]	-
			1.91 (0.77–3.93)	
omphalocele	[3]	[11]	-	-
	1.4 (0.4–4.5)	**3.2 (1.6–6.1)**		
gastroschisis	-	[11]	[4]	[6]
		1.3 (0.7–2.5)	2.26 (0.62–5.79)	**5.7 (1.8–15.9)**
Diaphragmatic hernia	[6]	[10]	[2]	[2] -
	1.8 (0.7–4.2)	1.7 (0.8–3.3)	0.63 (0.08–2.26)	
Oesophageal atresia	-	[9]	[3]	[0] -
		1.5 (0.7–3.0)	0.87 (0.18–2.55)	
Anal atresia	[7]	[8]	[6]	[1] -
	1.9 (0.8–4.3)	1.0 (0.4–2.0)	1.08 (0.40–2.36)	
Renal collecting system	[17]	-	-	-
	1.1 (0.7–1.9)			
Cystic kidney	-	-	[9]	-
			1.81 (0.83–3.44) **	
Hypospadias	[14]	[14]	[50]	[7]
	1.2 (0.6–2.1)	0.8 (0.4–1.5) *	**1.34 (1.01–1.78)** ***	2.3 (0.7–7.9) *
Undescended testes	[11]	-	-	-
	1.3 (0.7–2.5)			
Clubfoot	[20]	-	[24]	-
	**2.2 (1.4–3.6)**		1.16 (0.76–1.76)	
Limb reduction defect	[9]	-	[3]	[3]
	1.7 (0.9–3.4)		0.43 (0.09–1.25)	2.1 (0.4–7.6)
transverse LRD	-	[8]	-	-
		1.1 (0.5–2.4)		

* only II–III degree hypospadias; ** all 9 cases exposed to SSRI (RR = **2.39, 1.09**–**4.54**); *** among them 38 exposed to SSRI (OR = 1.30, 0.94–1.80).

Many studies have demonstrated that newborns that have been exposed *in utero* for SSRI have an increased risk of morbidity. Exposure during the later part of pregnancy seems to be most important for these outcomes. Neonatal morbidity is so common after SSRI that also small studies are informative [95,124,125] and have demonstrated an increased risk for prematurity, admission to special neonatal care, poor neonatal adaptation including respiratory difficulties, low Apgar score, hypoglycaemia, feeding difficulties, and cerebral excitation. In a study of 558 infants exposed to SSRI a doubling of the risk or preterm birth and low birth weight was noted but no increase in the risk of intrauterine growth retardation [126]. There was an increased risk for respiratory problems, low Apgar score, and neonatal convulsions and also an increased risk which did not reach statistical significance for hypoglycaemia. In the later study by Reis and Källén [93] similar results were found after any antidepressant exposure (mainly SSRI) but now the risk for hypoglycaemia was statistically significant and also the risk for jaundice. 

Oberlander and co-workers [127] used Canadian health registers and studied the effect of antidepressants using propensity-score matched controls and found an increased risk for preterm birth and respiratory distress even when maternal illness severity was accounted for. In another paper [128] they found that duration of exposure rather than timing affected pregnancy duration, birth weight and frequency of neonatal respiratory problems. These authors found an effect of maternal illness but a stronger effect of antidepressant use. In contrast, Suri *et al*. [129] concluded that no effect of maternal depression was seen on gestational age at birth but an effect of the use of antidepressants. An earlier study from Sweden also found no effect of maternal antenatal depression or anxiety on the risk of premature birth or neonatal complications [130]. Haeys *et al*. [131] noticed an effect of antidepressant use on preterm birth and respiratory problems and also a significant increase of neonatal convulsions after exposure during the third trimester. 

Generally, the neonatal effects are transient. Persistent hypertension in the newborn (PPHN) is a rare complication, notably in term infants. An increased risk for PPHN after maternal use of SSRI after week 34 has been observed [132,133]. A Nordic study also found similar results [134]. So far this association has not been seen with TCA or SNRI/NRI.

In one study [135] a high percentage of infants whose mothers had used SSRI immediately before delivery showed a QT interval prolongation, which normalized at follow-up. The significance of this observation is unclear.

Pregnancy complications as a result of antidepressant and notably SSRI use have been described. An increased risk for preeclampsia, hyperemesis and intrapartum bleeding was found after late pregnancy use of antidepressants [8]. An association between use of SSRI and an increased risk of gestational hypertension and preeclampsia has been described [136] and a significant risk increase of postpartum haemorrhage after antidepressants [137].

**Table 33:** Number of infants exposed in early pregnancy for antidepressants or was born of women who filled prescriptions for antidepressants during the 2nd or 3rd trimester.

ATC	Drug name	Early exposure	Late exposure	ATC	Drug name	Early exposure	Late exposure
*Tricyclic antidepressants*				*Monamine oxidase* * Inhibitors*			
N06AA02	imipramine	10	-	N06AF03	phenelzine	1	-
N06AA04	clomipramine	1,391	145	N06AG02	moclobemide	47	13
N06AA06	trimipramine	20	3	*Tryptophan*			
N06AA07	lofepramine	6	-	N06AX02	tryptophan	1	-
N06AA09	amitryptiline	658	232	*SNRI/NR*			
N06AA10	nortryptiline	47	12	N06AX03	mianserin	135	47
N06AA11	protyrptiline	3	-	N06AX06	mefazodone	50	-
N06AA14	melitracen	1	-	N06AX11	mirtazapine	585	368
N06AA21	maprotiline	2	-	N06AX12	amfebutamone/bupropione	69	58
*SSRI*				N06AX16	venlafaxine	1,677	469
N06AB03	fluoxetine	2,879	1,071	N06AX18	reboxetin	63	22
N06AB04	citalopram	6,996	2,325	N06AX21	duloxetine	206	173
N06AB05	paroxetine	1,687	390	N06AX22	agomelatine	3	1
N06AB06	sertraline	6,691	2,994	*Unspecified antidepressants*			
N06AB08	fluvoxamine	49	7	N06A	unspecified antidepressant	40	-
N06AB10	escitalopram	935	524				
N06AB	unspecified SSRI	145	-				

SNRI/NRI = serotonin/noradrenalin receptor inhibitor; SSRI = selective serotonin receptor inhibitors.

#### 3.6.2. Data from the Swedish Medical Birth Register

**Figure 6:**
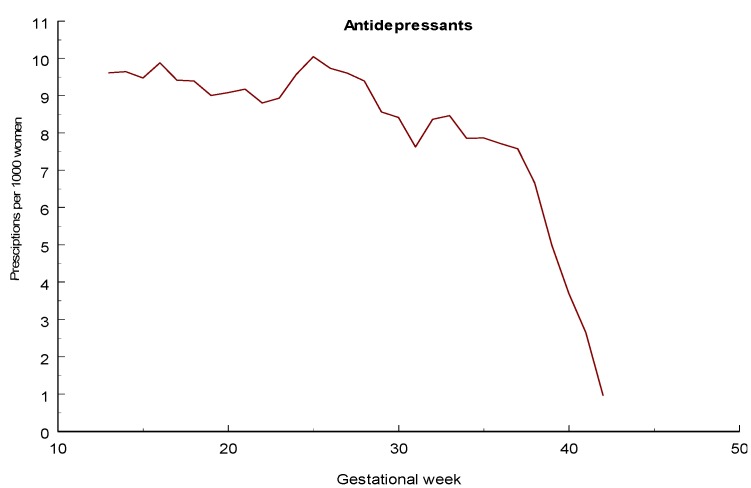
Diagram showing number of women per 1,000 who had filled a prescription for an antidepressant for each gestational week.

Table 33 lists the antidepressant drugs used. Antidepressant use in early pregnancy was reported by 23,342 women (23,658 infants). Among them 2,104 women (2139 infants) used TCA, 18,933 women (19,181 infants) used SSRI and 2,707 women (2,745 infants) used SNRI/NRI The total number of women who got prescriptions for antidepressants during the 2nd or 3rd trimester is 7,940 (8,053 infants). Among them, 387 women (394 infants) filled prescriptions for TCA, 6,919 women (7,014 infants) prescriptions for SSRI, and 1,050 women (1,071 infants) prescriptions for SNRI/NRI. As seen in Figure 6 there is a decline towards the end of the pregnancy in the number of prescriptions filled each pregnancy week. 

Table 34 updates the Reis-Källén [93] data on congenital malformations up to and including 2011. For all antidepressants analyzed together, there are no indications of a teratogenic effect. Table 35 describes the specific relationships between different groups of antidepressants or (when numbers are enough) specific antidepressants and different malformation groups. Statistically significant effects are seen only for clomipramine and for paroxetine (all cardiovascular defects and septum defects). There is also a high OR for hypospadias after paroxetine but significance is not reached.

**Table 34:** Congenital malformations in infants whose mothers used antidepressants in early pregnancy.

Malformation	Number with drug	Total number malformed	OR/RR	95% CI
All	1,048	70,339	0.86	0.81–0.91
Relatively severe	795	48,499	0.96	0.90–1.03
Chromosome anomaly	53	2,932	0.95	0.72–1.25
Neural tube defects	7	734	0.60	0.29–1.26
Other CNS malformations	11	1,131	0.67	0.37–1.20
Severe eye malformations	7	579	0.77	0.31–1.58
Severe ear malformations	0	280	-	-
Orofacial clefts	42	2,756	0.86	0.64–1.16
Any cardiac defect	283	16,145	1.00	0.89–1.12
Septal cardiac defect	208	11,157	1.04	0.91–1.20
Esophageal atresia	5	445	0.70	0.23–1.63
Small gut atresia	5	392	0.69	0.23–1.02
Anal atresia	12	590	1.18	0.66–2.10
Pyloric stenosis	24	1,101	1.14	0.77–1.70
Abdominal wall defect	9	413	1.25	0.57–2.37 #
Diaphragmatic hernia	5	368	0.86	0.28–2.00 #
Hypospadias	77	4,552	0.97	0.77–1.22
Major renal malformations	15	882	1.04	0.32–1.23
Pes equinovarus	45	2,127	1.15	0.75–1.56
Poly– or syndactyly	47	3,084	0.88	0.66–1.18
Limb reduction defects	12	830	0.86	0.49–1.52
Craniostenosis	15	862	1.10	0.65–1.83

# RR from observed/expected numbers with exact 95% CI based on Poisson distributions.

**Table 35:** Associations between specific antidepressant groups or drugs and some groups of malformations. Bold text marks statistical significance.

Malformation	Drug		Malformed with drug	Total number exposed	OR	95% CI
Relatively severe	Tricyclic		92	2,139	1.09	0.88–1.34
		clomipramine	69	1,399	1.20	0.94–1.53
	SSRI		627	19,181	0.94	0.87–1.01
		fluoxetine	102	2,879	0.92	0.81–1.05
		citalopram	229	6,996	0.94	0.83–1.07
		paroxetine	70	1,687	1.11	0.87–1.40
		sertraline	200	6,690	0.91	0.79–1.04
		escitalopram	32	935	1.10	0.70–1.71
	other antidepressants		92	2,745	1.00	0.81–1.24
		mirtazapine	20	585	1.10	0.70–1.71
		venlafaxine	60	1,577	1.05	0.81–1.36
		duloxetine	7	286	0.80	0.32–1.64 #
Any cardiac defect	Tricyclic		36	2,139	1.29	0.93–1.79
		clomipramine	28	1,399	**1.49**	**1.03–2.15**
	SSRI		211	19,181	0.92	0.81–1.05
		fluoxetine	41	2,879	1.03	0.81–1.32
		citalopram	65	6,996	0.80	0.63–1.02
		paroxetine	35	1,687	**1.63**	**1.17–2.27**
		sertraline	62	6,690	0.82	0.64–1.05
		escitalopram	9	935	0.92	0.47–1.77 #
	Other antidepressants		30	2,745	1.18	0.85–1.64
		mirtazapine	7	585	1.06	0.42–2.19 #
		venlafaxine	26	1,577	1.28	0.87–1.89
		duloxetine	2	286	–	–
Septum defects	Tricyclic		31	2,139	**1.66**	**1.17–2.35**
		clomipramine	23	1,399	**1.85**	**1.23–2.76**
	SSRI		151	19,181	0.94	0.80–1.14
		fluoxetine	27	2,879	0.93	0.68–1.29
		citalopram	47	6,996	0.83	0.62–1.11
		paroxetine	24	1,687	**1.67**	**1.12–2.50**
		sertraline	48	6,690	0.90	0.67–1.19
		escitalopram	6	935	0.85	0.31–1.84 #
	Other antidepressants		28	2,745	1.19	0.82–1.74
		mirtazapine	6	585	1.22	0.45–2.65 #
		venlafaxine	17	1,577	1.14	0.71–1.85
Hypospadias	Tricyclic		10	2,139	1.38	0.66–2.54 #
	SSRI		61	19,181	0.94	0.73–1.21
		paroxetine	10	1,687	1.69	0.81–3.12 #
		other SSRI	52	17,494	0.88	0.67–1.16
	Other antidepressants		10	2,745	1.14	0.55–2.10 #

# RR from observed/expected numbers with exact 95% CI based on Poisson distributions.

A combined use of different antidepressants occurred in 794 cases. Among them 29 infants had a “relatively severe malformation” (OR = 1.11, 95% CI 0.76–1.62) and seven had a cardiovascular defect (RR = 0.73, 95% CI 0.30–1.51). This material does not indicate an increased malformation risk after combined use of different antidepressants which could suggest a more severe psychiatric situation.

Table 36 describes the outcomes after the use of any antidepressant during the 2nd or 3rd trimester and Table 37 compares these outcomes for each one of the three major groups of drugs. There was an increased risk for preeclampsia which seemed to be stronger for TCA and SNRI/NRI than for SSRI. No effect is seen on gestational diabetes or placental abruption but an increased risk for haemorrhage around the delivery. The latter effect is similar for all three drug categories. Induction of delivery was undertaken more often than expected, notably after the use of TCA or SNRI/NRI but the risk after SSRI is also significantly increased.

**Table 36:** Outcomes after maternal use of antidepressants during the 2nd or 3rd trimester. Bold text marks statistical significance.

Outcome	With anti–depressant	In population	OR	95% CI
Preeclampsia	416	25,688	**1.27**	**1.14–1.40**
Gestational diabetes	124	7,118	1.13	0.94–1.36
Abruption of placenta	34	2,379	1.05	0.75–1.48
Haemorrhage around delivery	748	46,981	**1.32**	**1.22–1.42**
Induction of delivery	1,232	81,793	**1.18**	**1.11–1.26**
Preterm birth < 32 weeks	55	4,881	0.84	0.64–1.11
Preterm birth < 37 weeks	513	31,394	**1.29**	**1.17–1.41**
Low birth weight < 2500 g	328	20,712	**1.20**	**1.07–1.34**
Small for gestational age	224	16,440	1.02	0.89–1.17
Large for gestational age	293	19,745	1.11	0.99–1.25
Neonatal diagnosis	1,168	67,057	**1.41**	**1.32–1.50**
Respiratory diagnoses	575	27,708	**1.68**	**1.54–1.83**
Hypoglycaemia	275	14,455	**1.41**	**1.25–1.60**
Jaundice	348	26,928	0.98	0.88–1.10
CNS diagnoses	107	6,940	**1.23**	**1.02–1.50**
Low 5 min Apgar score	257	13,211	**1.52**	**1.34–1.72**

**Table 37:** Outcomes for three different groups of antidepressants: TCA, SSRI and SNRI/NRI. Bold text marks statistical significance.

	TCA			SSRI			SNRI/NRI		
Outcome	No.	OR	95% CI	No.	OR	95% CI	No.	OR	95% CI
Preeclampsia	23	1.41	0.91–2.18	329	**1.13**	**1.01–1.27**	87	**2.19**	**1.76–2.74**
Haemorrhage	35	1.24	0.88–1.76	650	**1.31**	**1.21–1.42**	102	**1.43**	**1.16–1.75**
Induction	79	**1.60**	**1.25–2.05**	1,015	**1.11**	**1.04–1.19**	220	**1.66**	**1.43–1.93**
<37 weeks	41	**2.11**	**1.54–2.91**	423	**1.22**	**1.10–1.34**	84	**1.56**	**1.24–1.95**
<2500 g	22	**1.58**	**1.03–2.44**	275	**1.16**	**1.03–1.31**	47	1.21	0.90–1.63
SGA	12	1.03	0.58–1.84	188	0.99	0.85–1.15	35	1.14	0.89–1.60
LGA	15	1.05	0.62–1.78	250	1.09	0.96–1.25	47	**1.39**	**1.03–1.88**
Neonatal diagnosis	77	**1.87**	**1.46–2.39**	1,000	**1.38**	**1.29–1.48**	159	**1.41**	**1.19–1.68**
Respiratory diagnosis	32	**1.79**	**1.25–2.56**	514	**1.73**	**1.58–1.89**	64	**1.35**	**1.05–1.75**
Hypoglycaemia	21	**2.04**	**1.33–3.15**	229	**1.36**	**1.19–1.55**	38	**1.41**	**1.02–1.96**
Jaundice	19	1.09	0.69–1.73	294	1.01	0.90–1.14	56	1.27	0.97–1.67
CNS diagnosis	8	1.85	0.70–3.64#	89	1.23	0.99–1.52	18	**1.62**	**1.01–2.59**
Low Apgar score	18	**2.07**	**1.23–3.27#**	226	**1.54**	**1.34–1.76**	30	1.32	0.92–1.91

# RR from observed/expected numbers with exact 95% CI based on Poisson distributions.

Among neonatal outcomes, an increased risk for preterm birth was seen but not for very preterm birth. Also low birth weight was increased in frequency but no certain effect was seen on SGA or LGA, but after exposure to SNRI/NRI a significant increased risk for LGA was found. There was an excess of neonatal morbidity which was seen for low Apgar score, respiratory and CNS diagnoses. The risk estimate for neonatal morbidity was higher after TCA than after SSRI or SNRI/NRI and the difference between TCA and SSRI seemed not to be random.

Few drug categories have been studied as extensively as antidepressants and notably SSRI. Some of the studies which seem to demonstrate teratogenic effects are retrospective case-control studies which may introduce methodological errors. In prospective studies little evidence of teratogenicity is found but the use of clomipramine or paroxetine may have a specific effect on cardiovascular defects, notably cardiac septum defects. When a pregnancy is planned, these drugs may be avoided but exposure for them is no reason for interrupting the pregnancy for fear of teratogenesis - the individual risk is low. Relatively little is known about other antidepressants than tricyclic or SSRI drugs but no major risk appears to exist.

Use of antidepressants during the 2^nd^ or 3^rd^ trimester is associated with a number of pregnancy and neonatal complications which usually are of temporary nature but will increase the need for neonatal intensive care. Data in the literature indicate that these effects may only partly be due to the drugs but partly is a result of underlying disease, a confounding by indication. There is a tendency that TCA causes stronger effects than SSRI at least for preterm birth, low birth weight and neonatal diagnoses which supports a drug effect but there may exist differences in underlying pathology which may contribute. These neonatal effects may indicate an increased risk for abnormal later development. The most serious neonatal complication is made up of PPHN which is rare and seems to be linked specifically to SSRI.

### 3.7. Psychostimulating Drugs

#### 3.7.1. Literature Review

This category of drugs has been used in different circumstances. Abuse is a common situation, often combined with other substance abuse including alcohol and smoking. Case reports of malformations after such exposures exist but no consistent finding of an increased malformation rate. Other perinatal and long term effects of maternal abuse of such drugs are well known. A risk increase of preterm birth, low birth weight and small-for gestational age is seen in infants whose mothers abused amphetamine (review in [138]). Also abuse of methylphenidate resulted in a high rate of preterm birth and growth retardation [139].

Another use of such drugs was as anorexiants. Case reports of congenital malformations in infants of mothers using dexamphetamine or phenmetrazine exist but larger series could not demonstrate a teratogenic risk but numbers of exposed infants were low [15]. These drugs are no longer used as anorexiants.

The present medical use is for behavioural diseases like attention deficit/hyperactivity disorder (ADHD) and the drug of choice in Sweden is methylphenidate. In a review [140] data for 180 infants exposed in utero for methylphenidate (104 of them from the Swedish Medical Birth Registry) were summarized with four malformations, all cardiovascular defects.

#### 3.7.2. Data from the Swedish Medical Birth Register

Only few exposures for such drugs were identified (Table 38). The largest group was methylphenidate followed by amphetamine. Abuse of amphetamine may be severely under-reported.

The OR for any congenital malformation after the use of any psychostimulant was 0.80 (95% CI 0.51–1.25), based on 19 exposed infants. Among them 13 had a “relatively severe malformation”, OR = 0.96 (95% CI 0.90–1.03). Five of these cases had cardiovascular defects (RR = 0.90, 95% CI 0.29–2.11), four of them had isolated VSD.

**Table 38:** Number of infants exposed in early pregnancy to psychostimulants.

ATC	Drug name	Early exposure	Late exposure
N06BA01	amphetamine	132	2
N06BA02	dexamphetamine	7	4
N06BA03	metamphetamine	1	-
N06BA04	methylphenidate	208	98
N06BA07	modafinil	19	13
N06BA09	atomoxetine	22	12
N06BC01	caffeine	60	61
N06BX03	piracetam	2	-
N06BX13	idebenone	1	1

For amphetamine (132 exposures) there was one multi-malformed infant with an unspecified intestinal malformation, lobster-claw hand and unilateral radial reduction. The mother had also used haschish, oxazepam and flunitrazepam. Another three infants had isolated malformations (pes equinovarus, hydronephrosis or pyloric stenosis).

After early pregnancy use of methylphenidate (208 exposures) there were five infants with relatively severe malformations. All five had cardiovascular defects. One infant had a d-transposition with double outlet right ventricle, aortic atresia and VSD. The other four infants all had VSD. The RR for a cardiac defect was 1.81 (95% CI 0.59–4.21).

Only 180 women with 184 infants had got prescriptions for psychostimulators and for 60 of them it referred to caffeine (Table 38). This small material has a low power to demonstrate associations with outcome (Table 39). When the analysis was restricted to three related drugs used at ADHD and similar conditions (methylphenidate, modafinil, atomoxetine, 122 infants) the risk for preterm birth reached statistical significance (OR = 1.83, 95% CI 1.00–3.36) and the risk for neonatal morbidity was RR = 1.50 (95% CI 0.92–2.46), thus not quite statistically significant.

**Table 39:** Outcomes after maternal use of psychostimulants during the 2nd or 3rd trimester.

Outcome	With psycho–stimulants	In population	OR	95% CI
Preeclampsia	8	25,688	1.01	0.44–1.99 #
Gestational diabetes	2	7,118	-	-
Abruption of placenta	1	2,379	-	-
Haemorrhage	17	46,981	1.40	0.85–2.31
Induction of delivery	28	81,793	1.13	0.75–1.71
Preterm birth < 37 weeks	15	31,394	1.43	0.84–2.46
Low birth weight < 2500 g	7	20,712	0.92	0.37–1.90
Neonatal diagnosis	24	67,057	1.16	0.75–1.71
Respiratory diagnoses	9	27,708	1.08	0.50–2.06 #
CNS diagnoses	4	6,940	2.15	0.59–5.51 #
Low 5 min Apgar score	3	13,211	0.70	0.15–2.13

# RR from observed/expected numbers with exact 95% CI based on Poisson distributions.

No strong teratogenicity seems to exist for psychostimulants but enough data are not available yet. There are weak indications that use of methylphenidate causes an increased risk for cardiovascular defects but further studies of this possible association are needed.

The risk associated with abuse of psychostimulating drugs like amphetamine is well known even though often mixed abuse situations may exist. These drugs are used therapeutically mainly at ADHD and related conditions. Available data for this situation are largely missing. There was a marginally increased risk for preterm birth but not for low birth weight which indicates an increased probability for LGA. There were also signs of an increased risk of neonatal morbidity but statistical significance was not reached. Further data are needed before a final risk evaluation can be made.

### 3.8. Other CNS-Active Drugs

#### 3.8.1. Literature Review

The relation between maternal smoking and congenital malformations has been studied extensively and some associations appear to be well demonstrated, e.g., with orofacial cleft. Smoking, however, represents much more than nicotine exposure. An exposure which would selectively identify nicotine effects would be use of nicotine patches or other similar substitutes for smoking (NRT). Only little information on this is available in the literature. A just statistically significant increase in malformation risk among non-smokers using nicotine substitutes has been described, notably for musculoskeletal malformations [141]. Most other studies on nicotine replacement treatments refer to other pregnancy outcomes than congenital malformations. Nicotine replacement therapy (NRT) has been associated with an increased rate of low birth weight and preterm birth, not only compared with non-smokers but also with smokers [142]. A Danish study found no indication of an increased stillbirth risk after NRT [143] and a USA study found no increased risk for adverse birth and neonatal outcomes when the confounding from previous preterm births was adjusted for [144].

Data on the possible effect on congenital malformations of treatment of opioid-dependent women during pregnancy are also few and based on low numbers. A comparison was made of the outcome after treatment with buprenorphine or methadone [145]. There was no difference in malformation frequency but this finding is rather uncertain due to low numbers (86 and 40 live births, respectively). Other studies have identified neonatal effects of use of methadone [146,147,148,149] and small comparative studies between buprenorphiine and methadone have suggested less severe effects of the former drug [150].

**Table 40:** Number of infants exposed in early pregnancy to “other CNS-active drugs” or were born to women who filled prescriptions for such drug during then 2nd or 3rd trimester.

ATC	Drug name	Early exposure	Late exposure
N06D	*Drugs for dementia*		
N06DA03	rivastigmine	2	-
N07A	*Parasymphatomimetics*		
N07AA01	neostigmine	1	-
N07AA02	pyridostigmine	63	16
N07AA30	ambenonium	3	2
N07AB01	carbachol	1	2
N07AB03	acetylcholine	1	6
N07AX01	pilocarpine	3	-
N07B	*Drugs for dependence*		
N07BA01	nicotine	203	19
N07BA02	bupropion	35	2
N07BA03	verenicline	25	92
N07BB01	disulfiram	13	12
N07BB03	acamprosate	2	-
N07BB04	naltrexone	1	3
N07BC01	buprenorphine	98	50
N07BC02	methadone	20	21
N07BC51	buprenorphine combination	6	14
N07C	*Drugs for vertigo etc.*		
N07CA01	betahistine	3	2
N07CA02	cinnarizine	134	-
N07CA03	flunarizine	1	1
N07X	*Drugs for ALS*		
N07XX02	riluzole	1	22
N07	*Unspecified*	3	1

#### 3.8.2. Data from the Swedish Medical Register

Table 40 lists the drugs included under this heading. It is a mixture of different drug categories. Parasympathomimetics with pyridostigmine as the most common one are used at myasthenia gravis and intestinal atonia. Among drugs for treating dependence, nicotine is certainly strongly under-reported. Such drugs are available as OTC, also in grocery stores, and are perhaps not even always regarded as drugs. Buprenorphine is used at opioid dependency. The dominating drug at vertigo is cinnarizine, an antihistamine also used for nausea.

Antismoking treatment mainly takes place with nicotine replacement which is sold without prescription and therefore relatively few women who filled prescriptions for such drugs were identified.

After early exposure to any of these drugs, 21 infants had a “relatively severe” malformation, OR = 0.82, 95% CI 0.53–1.26. After nicotine the RR was 0.96 (95%CI 0.45–1.66) based on nine exposed malformed infants, after buprenorphine 1.01 (95% CI 0.21–2.66) based on three exposed malformed infants, and after cinnarizine 0.80 (95% CI 0.26–1.88) based on five exposed malformed infants.

The risk for preterm birth was not increased in any of the dependence drugs. The RR for the largest group (NRT) was 0.71 (95% CI 0.24–1.70). The risk for neonatal morbidity was increased for NRT but did not reach statistical significance: OR = 1.39, 95% CI 0.83–2.34). After the use of drugs for alcohol abuse, only two infants showed neonatal morbidity (expected number 2.3) but after the use of drugs for opioid abuse, the OR was significantly increased: RR =1.92 (95% CI 1.10–3.12).

Drugs used for treatment of opioid abuse may be associated with an increased risk for neonatal problems and notably CNS effects. If this is due to the treatment or to the underlying abuse situation is difficult to disentangle. Too little information is available on the effect of NRT but the estimated ORs for neonatal morbidity and notably for CNS problems are increased although not statistically significant. Further data are needed.

### 3.9. Use of more than one Group of CNS-Active Drugs

#### 3.9.1. Literature Review

Even though it has repeatedly been acknowledged that women using CNS active drugs often use combinations of drugs with different effects, e.g., sedatives/hypnotics and antidepressants, data on the possible additive or synergistic effects in such combinations are seldom investigated. One example is a study which claimed that use of SRI drugs did not increase malformation risk, neither did the use of benzodiazepines, but the combined use of both drug categories carried a risk, notably for cardiovascular defects [117]. A recent study could not verify the finding [151].

Relatively little data are available on possible synergistic or additive effects on the neonatal outcome when different CNS active drugs are used concomitantly. In a previous study [25] based on about half of the present material, the combination of SSRI and other CNS active drugs was explored and it was found that such combinations resulted in more severe disturbances of the neonatal outcome than when the drugs were used alone. Another example of an effect of concomitant drug use was described for women using methadone – the strength and frequency of neonatal abstinence syndrome was influenced by simultaneous use of opioids, benzodiazepines or cocaine [152].

#### 3.9.2. Data from the Swedish Medical Birth Register

The number of infants born of women who reported the use of two or more different categories of CNS active drugs during early pregnancy or had filled prescriptions for two or more CNS-active drugs during the 2nd or 3rd trimester is shown in Table 41. It is not certain that the two drugs were used simultaneously. Women who used dixyrazine or prochlorperazine are excluded from the neuroleptics group.

**Table 41:** Cross-tabulation of CNS active drugs: number of infants exposed in early pregnancy or born by women who filled prescriptions for two or more CNS active drugs during the 2nd or 3rd trimester. Neuroleptics exclude dixyrazine and prochlorperazine. The first column gives the explanation to the column ATC codes.

Drug	Alone	N03	N04	N05A	N05B/C	N06A	N06B	N07
*Early drug use*								
Opioids (N02A)	6,734	107	16	48	486	562	18	17
Anticonvulsants (N03)	3,799	-	7	109	221	374	24	17
Antiparkinson (N04)	84	-	-	40	23	38	0	0
Neuroleptics (N05A)	3,343	-	-	-	326	488	17	6
Sedatives/hypnotics (N05B/C)	4,158	-	-	-	-	2,379	79	38
Antidepressants (N06A)	20,165	-	-	-	-	-	91	58
Psychostimulants (N06B)	171	-	-	-	-	-	-	8
*Drug use 2nd or 3rd trimester*								
Opioids (N02A)	11,739	117	24	59	1,283	1,157	5§	48
Anticonvulsants (N03)	805	-	14	68	248	216	12	12
Antiparkinson (N04)	18	-	-	23	32	32	1	0
Neuroleptics (N05A)	114	-	-	-	281	235	16	0
Sedatives/hypnotics (N05B/C)	2,998	-	-	-	-	2,226	55	46
Antidepressants (N06A)	5,028	-	-	-	-	-	39	45
Psychostimulants (N06B)	84	-	-	-	-	-	-	7
Other CNS-active drugs (N07)	131	-	-	-	-	-	-	

Table 42 shows the presence of congenital malformations after combined used of CNS-active drugs in early pregnancy. There is one combination which shows a significant over-risk: benzodiazepines and antidepressants with “relative severe malformations” as outcome – while the combination HBRA and antidepressants with the same outcome showed a significant protective effect. Both may be explained by multiple comparisons. The first association is strongest for tricyclic antidepressants which may have an effect of their own (see above).

An increased RR which did not reach statistical significance was seen for the combination of benzodiazepines and SSRI with regard to cardiac defects, resembling the combination which was identified by Oberlander *et al*. [117]. Table 43 specifies the drugs and malformations among the eleven malformed infant who were exposed to this combination of drugs. When the RR for this combination (1.47, 95% CI 0.73–2.62) was compared with the OR for a cardiovascular defect after exposure to benzodiazepines but not SSRI (1.34, 95% CI 0.98–1.82) and that for SSRI without exposure to benzodiazepines (0.92, 95% CI 0.81–1.05) it was evident that these variations in risk estimates can be random, notably for benzodiazepines with or without SSRI.

**Table 42:** “Relatively severe malformations” and cardiovascular defects after combined use of different CNS active drug groups. Neuroleptics exclude dixyrazine and prochlorperazine. Bold text marks statistical significance.

Drug combination	Number. malformed	Total number	OR/RR	95% CI
*Relatively severe malformations*				
Opioids+anticonvulsants	5	107	1.37	0.44–3.19 #
Opioids+sedative/hypnotics	13	486	0.75	0.44–1.29
Opioids+antidepressants	21	562	1.09	0.71–1.68
Anticonvulsants+neuroleptics	6	109	1.76	0.65–3.83 #
Anticonvulsants+sedatives/hypnotics	9	221	1.13	0.52–2.15 #
Anticonvulsants+antidepressants	15	374	1.02	0.62–1.67
Neuroleptics+sedatives/hypnotics	16	326	1.23	0.76–2.00
Neuroleptics+antidepressants	16	488	1.16	0.71–1.91
Sedatives/hypnotics+antidepressants	87	2,379	0.95	0.79–1.15
Benzodiazepines + antidepressants	43	902	**1.36**	**1.01–1.83**
Benzodiazepines + tricyclic	9	130	1.82	0.83–3.45 #
Benzodiazepines + SSRI	29	651	1.26	0.87–1.81
Benzodiazepines + SNRI/NRI.	4	163	0.77	0.21–1.97
*Any cardiovascular defect*				
Opioids+sedative/hypnotics	3	486	0.49	0.10–1.44 #
Opioids+antidepressants	8	562	1.23	0.53–2.43 #
Anticonvulsants+neuroleptics	2	109	-	-
Anticonvulsants+sedatives/hypnotics	5	221	1.95	0.63–4.56 #
Anticonvulsants+antidepressants	8	374	1.92	0.85–3.78 #
Neuroleptics+sedatives/hypnotics	5	280	1.18	0.38–2.76 #
Neuroleptics+antidepressants	3	402	0.62	0.13–1.80 #
Sedatives/hypnotics+antidepressants	23	2,379	0.76	0.52–1.11
Benzodiazepines + antidepressants	14	902	1.30	0.77–2.20
Benzodiazepines + tricyclic	2	130	-	-
Benzodiazepines + SSRI	11	651	1.47	0.73–2.62 #
HBRA+antidepressants	3	163	**0.32**	**0.07–0.94 #**
Hydroxyxine+antidepressants	4	393	0.95	0.26–2.42 #
Propiomazine+antidepressants	4	686	0.39	0.15–1.00

# RR from observed/expected numbers with exact 95% CI based on Poisson distributions.

Among women who had filled prescriptions for two or more different CNS active drug groups during the 2nd or 3rd trimester, there are seven combinations with more than 200 women involved – these have been analyzed separately and the outcomes are summarized in Table 44 This Table shows that relatively high odds ratios were obtained for the outcomes studied in many combinations. Some of them are based on small numbers why the confidence intervals are wide, e.g., the high odds ratio for CNS diagnosis after combined use of anticonvulsants and sedative/hypnotics. 

**Table 43:** Eleven cases with cardiovascular defects after maternal use of benzodiazepines and SSRI.

Case No.	Benzodiazepine	SSRI	Other CNS drug	Heart defect	Other defect
1–2 (twins)	lorazepam	paroxetine	-	VSD	hypospadias
3	alprazolam	sertraline	-	VSD+ASD	-
4	alprazolam	citalopram	-	pulmonary valvestenosis	-
5	oxazepam	paroxetine	-	pulmonary valvestenosis	-
6	oxazepam	escitalopram	-	VSD+ASD	-
7	oxazepam	sertraline	zoplicone	CoA	-
8	diazepam	sertraline	-	VSD	-
9	oxazepam	fluoxetine+citalopram	pregabalin	VSD+ASD	-
10	oxazepam	fluoxetine	clonazepam	Ebstein’s anomaly	-
11	alprazolam	fluoxetine	-	Fallot’s tetralogy	-

**Table 44:** Some outcomes for different combinations of CNS-active drugs. Odds ratios given with 95% confidence intervals. Bold text marks statistical significance. For number of exposed infants, see Table 41.

Drug 1	Drug 2	<37 weeks	Neonatal Diagnosis	Respiratory diagnosis	CNS diagnosis
Opioid	Antidepressant	**1.87 (1.53–2.29)**	**1.66 (1.43–1.94)**	**1.95 (1.59–2.39)**	**1.58 (1.02–2.46)**
Opioid	Sedative/ hypnotic	**2.26 (1.85–2.76)**	**1.73 (1.55–1.94)**	**1.92 (1.64–2.24)**	**1.86 (1.37–2.53)**
Sedative/ Hypnotic	Antidepressant	**1.67 (1.38–2.03)**	**1.71 (1.54–1.91)**	**1.94 (1.67–2.25)**	**1.64 (1.19–2.26**
Anti- convulsant	Antidepressant	**2.40 (1.58–3.64)**	**2.07 (1.50–2.85)**	**2.00 (1.26–3.17)**	2.11 (0.69–4.92) #
Anti- convulsant	Sedative/ hypnotic	**2.09 (1.75–2.50)**	**1.76 (1.28–2.42)**	**1.78 (1.14–2.80)**	**3.90 (2.01–6.81) #**
Neuroleptic *	Sedative/ hypnotic	1.25 (0.79–1.98)	**1.57 (1.15–2.14)**	**1.90 (1.26–2.85)**	2.27 (0.98–4.48) #
Neuroleptic *	Antidepressant	1.56 (0.96–2.41)	**1.69 (1.20–2.36)**	**1.92 (1.64–2.24)**	1.66 (0.54–3.86) #

* excluding dixyrazine and prochlorperazine; # Relative risk as observed over expected number with 95% CI from exact Poisson distribution.

**Figure 7:**
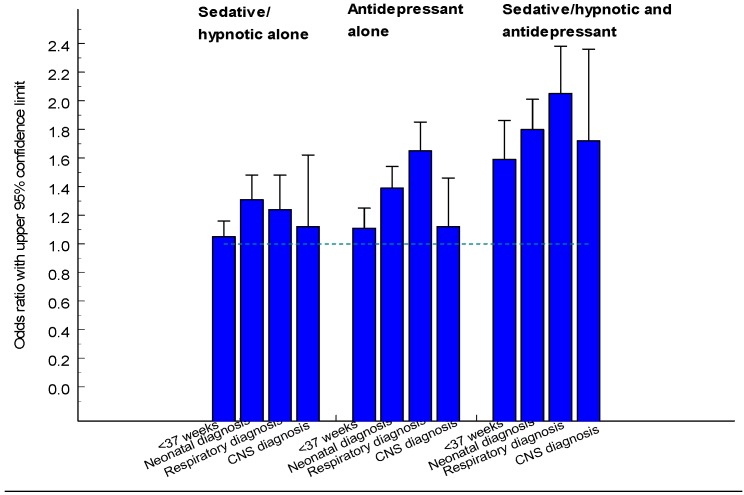
Diagrams showing odds ratios for four neonatal outcomes after maternal use of sedatives/hypnotics or antidepressants alone and after use of a combination of the drugs.

**Figure 8:**
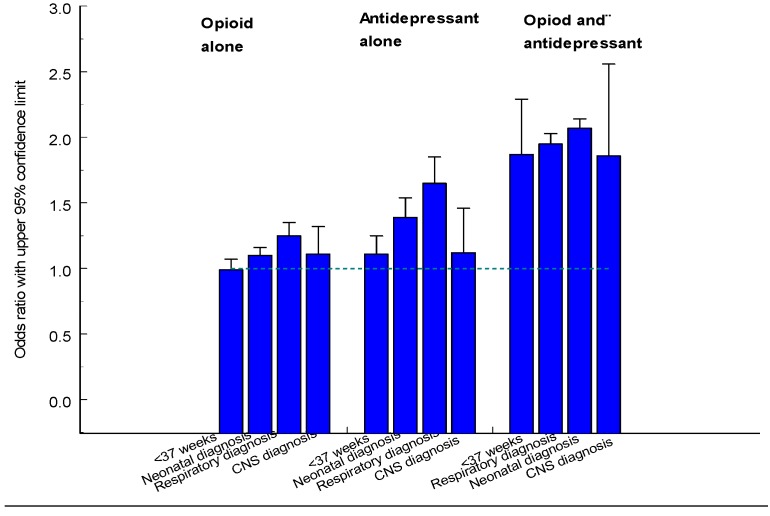
Diagrams showing odds ratios for four neonatal outcomes after maternal use of opioids or antidepressants alone and after use of a combination of the drugs.

**Figure 9:**
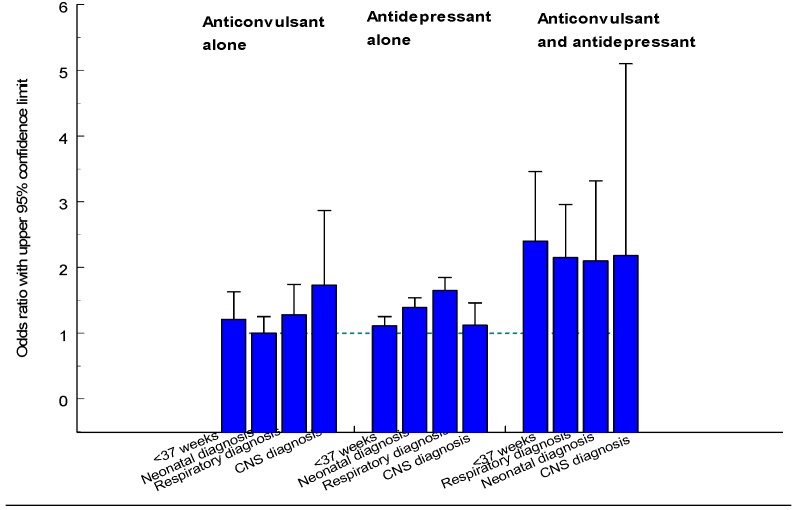
Diagrams showing odds ratios for four neonatal outcomes after maternal use of anticonvulsants or antidepressants alone and after use of a combination of the drugs.

These high ORs should be compared with ORs obtained for the components of the combination when these drugs were used alone. Such comparisons are made for three combinations with antidepressants (Figure 7, Figure 8 and Figure 9). For most of these combinations the impression is that the combined effect of the involved drugs is additive so no true synergism occurs. For preterm birth, however, the combined effect for all three combinations is markedly higher than the sum of the effects of the involved drugs. If this is the result of synergism or is a result of confounding by more severe underlying disease is difficult to say.

Use of a combination of CNS active drugs is relatively common. So far no synergism seems to exist between different types of CNS active drugs in early pregnancy with respect to teratogenesis. Numbers of exposures for many combinations are small and weak synergisms might have been missed in the analysis.

The neonatal outcome after combined exposures during the 2^nd^ or 3^rd^ trimester is worse than after single drug exposures. For most neonatal morbidity it seems as if the effects are additive but for preterm birth they seem to be more than additive. This could be a result of synergism but could also be due to more severe underlying pathology which motivated a more complex drug therapy.

## 4. Long Term Effects of Intrauterine Exposure to CNS-Active Drugs

### 4.1. Literature Review

The possibility that exposure during pregnancy for drugs and notably CNS active drugs could have long-time effects on neuropsychiatric development and perhaps have other late consequences has been realized for a long time and extensive animal research have demonstrated the biological plausibility of such effects. To demonstrate such effects in the human is difficult, however. A relatively long follow-up is needed and to this is added the possibility that noted effects can have other explanations than direct drug effects on the developing brain. One possibility is a genetic explanation. Some neuropsychiatric conditions like autism and ADHD have a genetic component and if CNS active drugs are used for such conditions, a link with offspring pathology can be obtained. This has been demonstrated for the link between maternal smoking during pregnancy and an increased risk for ADHD in the offspring. This link has been described by many authors but recent studies have indicated that the explanation is that women who have signs of ADHD are more likely to smoke than other women [153] and studies on women who had egg donations found no effect of recipient smoking on the ADHD risk [154]. A further complication is of course the influence of the environment for the child whose mother has psychiatric problems. In the early studies on the neuropsychological development of children born of mothers who abused alcohol, the distinction between prenatal and postnatal effects could be solved by the study of children born to alcoholic mothers who had been adopted by non-alcoholic parents. They seemed to develop the same abnormalities as children who stayed in the family with its biological mother [155].

The serious effects of maternal abuse of various drugs on the neuropsychiatric development of the child are well known from many studies. Poor infant psychomotor development after maternal amphetamine [156], ecstasy [157], or methamphetamine [158,159] abuse is well known. A general effect of illicit drug use was found on oppositional defiant disorder and adult antisocial behaviour while an increased risk for ADHD did not reach statistical significance [160].

There are thus clear-cut ill effects of maternal drug abuse but the information on possible long-term neuropsychological disturbances as a result of medical treatments with CNS-active drugs are less well understood with the exception of the effects of anticonvulsants.

Already in the review by Bossi [55], studies were referred to which indicated long-term retardation of psychomotor development after anticonvulsants. Many later studies have demonstrated such effects. For instance early cognitive development has been studied [161] and a stronger effect on verbal than non-verbal abilities has been described [162,163]. Studies of IQ at 4.5 years showed a negative association with maternal valproic acid use but not with other anticonvulsants [164]. The marked effect of valproic acid was also evident from a meta-analysis [165]. Most of these studies consisted of small materials utilizing various forms of psychological tests.

One study compared school marks at the age of 16 (when children leave compulsory school) and found poorer school performance in children whose mothers had used anticonvulsants during pregnancy than in controls and found a stronger effect of polytherapy than of monotherapy [166]. Another population-based study found an increased risk of autism spectrum disorders and childhood autism after prenatal exposure to valproic acid while exposure to other anticonvulsants resulted in no significant effect [167].

Some data are also available on long-term outcomes after maternal use of antidepressants and notably SSRI drugs. An early study of restricted size [168] found no differences between an exposed group of children (tricyclic antidepressants or fluoxetine) and a control group with respect to global IQ at 16–86 months age, temperament, mood, arousability, activity level, distractibility or behaviour problems. Another small study investigated attentional and activity behaviours among 4-year old children after maternal use of SSRI without finding any differences from a small control group [169].

It has been suggested that serotonin plays a role in the origin of autism and that maternal use SSRI drugs therefore could affect the risk for autism in the child [170,171] and one study found twice as many SSRI users among mothers of 20 children with autism than among mothers of 50 control children [172]. Another study found that maternal (but not paternal) bipolar disorder, psychotic disorder or depressive disorder increased the risk for ADHD in the child and a strong association with the use of bupropion was found, notably during the second trimester [173]. 

A population-based study from Denmark found that children exposed to SSRI during the 2nd or 3rd trimester showed a delay in motor development compared with unexposed children and also that there was a difference in the ability to occupy themselves at the age of 19 months [174].

It is rather unlikely that small cohort studies can identify changes in the frequency of outcomes like autism or ADHD. Case-control studies will have a greater power but may introduce bias from the ascertainment of drug use. Population-based studies can overcome these problems but the complex confounding possibilities in studies of such outcomes remain and make conclusions uncertain. 

### 4.2. Data from the Swedish Medical Birth Register

Few studies have been made using this register on long term effects of drugs with the exception of the study mentioned above on anticonvulsants [166].

A previously unpublished study of the possible association between maternal use of antidepressants during pregnancy and child ADHD is presented here – not as a proof of a causality but to illustrate the problems in this type of studies.

We identified women who had reported the use of antidepressants in early pregnancy according to the Medical Birth Register for the years 1995–2006. We identified children who were likely to have ADHD from the Prescribed Drug Register during the years 2005–2008, using prescriptions for methylphenidate or atomoxetine as definitions – drugs which are relatively selective for the treatment of ADHD.

The study was based on a total of 1,132,442 live born children – among them 11,465 were identified as having drug treated ADHD (1.0%). Use of antidepressants in early pregnancy was reported by 10,442 women (0.9%). The selected group of children with ADHD probably represents relatively severe cases as they needed drug therapy. The group of women using antidepressants may be incomplete but as they were identified in early pregnancy their identification must be unbiased. 

A total of 185 ADHD children were born of mothers who had reported the use of antidepressants. A crude OR for ADHD after maternal use of antidepressants was 1.78 (95% CI 1.53–2.06). As use (or registration) of antidepressants increased with delivery year and at the same time the observation period for ADHD decreased, adjustment for year of birth resulted in an increase in OR to 2.59 (95% CI 2.24–2.98). A number of maternal characteristics can affect the risk for child ADHD and if also associated with maternal use of antidepressants they could confound the analysis. Further adjustment for maternal age, parity, maternal smoking in early pregnancy, pre-pregnancy BMI, subfertility, mother being born outside Sweden, cohabitation, and maternal education reduced the OR to 2.08 (95% CI 1.87–2.50). For a selected subgroup of women who were born in Sweden and were cohabiting and had an education of at least 14 years, the OR was higher: 3.79 (95% CI 2.45–5.75). If this analysis was restricted to singletons the OR increased slightly. 

There was no statistically significant difference between the ORs according to type of antidepressants (Table 45). The difference between the three main antidepressant groups was not significant (*p* = 0.12) but the difference between the four SSRI groups may be (*p* = 0.03), mainly due to the low OR for paroxetine.

**Table 45:** Adjusted OR for ADHD after maternal use of various types of antidepressants.

Antidepressant used	With ADHD and antidepressant	Total with antidepressant	OR	95%CI
TCA	35	1,678	1.81	1.30–2.52
Clomipramine	29	1,263	1.82	1.27–2.62
SSRI	138	8,875	2.37	1.89–2.65
Fluoxetine	27	1,300	3.79	2.61–5.47
Citalopram	64	3,518	2.07	1.62–2.65
Paroxetine	15	1,130	1.39	0.82–2.35
Sertraline	30	2,808	2.23	1.56–3.18
SNRI/NRI	13	1,138	2.02	1.18–3.48
Venlafaxine	7	726	1.89	0.91–3.92

These data show an association between maternal use of antidepressants and child ADHD but do not prove a direct drug effect. A possible confounding is present if women with features of ADHD – whose children have an increased risk for ADHD for genetic reasons - are more likely than other women to get antidepressant treatment. This possibility is difficult to adjust for in register studies. Causality is thus far from proved.

Long-term effects of drug exposures during pregnancy are of great importance but are also problematic to identify due to a number of confounders which are difficult to measure. For studies of less common outcomes like autism or ADHD relatively large studies are needed but analyses must take into consideration the heredity of such conditions. With the exception of abuse situations, no firm evidence exists of long-

term effects of exposure for CNS-active drugs but large, well-designed studies are needed to clarify these issues. Harmful such effects can be obtained from exposures during any time of pregnancy but probably the 2^nd^ and 3^rd^ trimesters are most vulnerable. Until better data have been obtained, exposure should be kept as low as possible with due consideration to the woman´s health.

## 5. Discussion and Conclusions

Women using CNS-active drugs during early pregnancy differ in many ways from other women, both with respect to maternal characteristics and concomitant drug use. Adjustment for maternal smoking has been made but information on alcohol use is not available. After adjustment for such factors, very little risk increase for infant congenital malformations can be seen with the exception of the well-known teratogenic properties of some anticonvulsant drugs. A number of possible associations between drug use and congenital malformations were apparent in the present analysis and from previous published studies which were based on good-quality data. In the following we will list these tentative or definite associations:

1. Tramadol and pes equinovarus. No similar effect is seen with other opioids. No support exists from the previous literature.
2. Anticonvulsants and a number of different malformation groups. There is a higher risk at polytherapy than at monotherapy, specifically linked to valproic acid but observable also with other anticonvulsants. This observation is well supported by previous literature. When possible, valproic acid should be avoided during pregnancy. Mounting evidence supports a teratogenic effect of topiramate but more data are needed. Carbamazepine or lamotrigine in monotherapy appear to be associated with a low malformation risk.
3. Among neuroleptics, flupentizol appears to be associated with an increased malformation risk, perhaps notably for urogenital malformations. This association has been indicated in previous studies from the Swedish Medical Birth Register but not by other studies. The old discussion on the possible teratogenic effect of lithium is still not definitely answered.
4. An association between use of benzodiazepine and pyloric stenosis is seen which has not been described from other sources. This observation needs confirmation.
5. Use of clomipramine or paroxetine is associated with an increased risk for cardiovascular defects and notably septum defects. The latter association has been observed in some but not all previous studies.
6. A “protective” effect for malformations is seen from the combination of HBRA and antidepressants and from the use of dixyrazine or prochlorperazine. The latter finding is explainable as a confounding by indication, NVP.

Many other associations have been described in the literature. Most of them are due to the methodological weaknesses of retrospective studies with high non-response rates. Such studies should not be undertaken when alternative methods exist. There are different possible explanations to the identified associations. One is that they are the result of multiple testing. A very large number of exposures and outcomes are compared and some will just by chance appear statistically significant, either as a risk factor or as a protective factor. Another explanation is that there is a confounding by indication, that the reason for a changed risk of a congenital malformation is the underlying disease. This may explain the “protective” effect of dixyrazine or prochlorperazine used at NVP. In some instances this explanation is less likely, namely, when the effect is seen in only one drug among many used at similar conditions. An example is paroxetine which in some studies are linked to an increased risk of a heart defect, not seen with other SSRI drugs. Another is the markedly higher teratogenic risk with valproic acid than with other anticonvulsants. Third, there may be causality so the drug actually causes the malformation. This has been suggested for instance for clomipramine where a mechanism has been proposed for the teratogenic action.

In order to eliminate or verify the first explanation (multiple testing) the only way is to repeat the study on fresh materials. It should also be stressed that multiple testing may also randomly hide a causal connection when by chance a low risk estimate has been obtained. 

It is a problem to translate the research findings into clinical practice. When a drug has been definitely linked with a teratogenic property (e.g., valproic acid) it is reasonable to try to avoid exposure during pregnancy if that is at all possible. If exposure has occurred, prenatal diagnosis may be intensified and in rare cases pregnancy interruption may be considered. When more uncertain associations exist, pregnancy interruption should not be considered and one should realize that even if a risk increase may exist it is usually moderate and will be of little concern for an individual woman. On the other hand, avoidance of such drugs during pregnancy can be recommended until the issue has been clarified. If possible, it could be reasonable to try to switch paroxetine to another SSRI when a pregnancy is planned, even though the link between paroxetine and cardiac defects is still debated

In the situation when a couple has had a malformed infant and the mother had used a drug with a suspected teratogenicity, the causality in the individual case depends on the size of the risk increase. Even if the risk is doubled, it is only 50% chance that the drug caused the malformation in the specific case.

Use of at least some CNS-active drugs during the late part of the pregnancy is associated with definite maternal and neonatal pathology. This is most obvious for sedatives and hypnotics and for antidepressants but can be seen also after anticonvulsants, antipsychotics and medical use of opioids. These effects will result in an increased need for neonatal care but in most instances the neonatal symptoms disappear within days or weeks. The possibility exists that the neonatal effects in the long term will increase the risk for developmental disturbances, notably of a neuropsychiatric nature, similar to those seen after maternal drug abuse. Enough high quality data are not avilable to evaluate such risks after medical use of these drugs. Such an analysis may also be confounded by the mother’s underlying disease. All this together makes it reasonable to try to keep exposure as low as possible without risking the well-being of the mother. 

## Abbreviations

AED: antiepileptic drug
ATC: Anatomical Therapeutic Chemical clssification of drugs
CNS: central nervous system
FGA: first generation antipsychotics
ICD: International Statistical Classification of Diseases and Related Health Problems
LGA: large for gestational age
LMP: last menstrual period
NSAID: no-steroid antiinflammatory drug
NVP: nausea and vomiting in pregnancy
PPHN: persistent pulmonary hypertension of the newborn
SD: standard deviation
SGA: small for gestational age or second generation antipsychotics
SNRI/NRI: serotonin-noradrenaline reuptake inhibitor or noradrenaline reuptake inhibitor
SSRI: selective serotonin reuptake inhibitor
TCA: tricyclic antidepressant
